# Pioneering biomimetic biomaterial for leukemia therapy enhancement: a review

**DOI:** 10.3389/fbioe.2025.1713597

**Published:** 2025-11-27

**Authors:** Xiang Yao, Zihao Wang, Yeke Zhang, Zhengyang Shao, Junlan Lian, Yue Chen, Xuanya Lin, Danping Qu, Wei Yang

**Affiliations:** 1 Department of Hemato-Oncology, Hangzhou Red Cross Hospital, Hangzhou, Zhejiang, China; 2 The Second Affiliated School of Zhejiang Chinese Medical University, Hangzhou, Zhejiang, China; 3 Department of Presbyatrics, Hangzhou Red Cross Hospital, Hangzhou, Zhejiang, China; 4 Department of Pediatrics, Hangzhou Red Cross Hospital, Hangzhou, Zhejiang, China; 5 Hubei University of Chinese Medicine, Wuhan, Hubei, China; 6 Department of Pediatrics, Yuhuan Traditional Chinese Medicine Hospital, Taizhou, Zhejiang, China

**Keywords:** biomimetic, biomaterial, leukemia therapy, targeted drug delivery, review

## Abstract

Leukemia is a highly recurrent and metastatic hematologic malignancy. The disease poses significant challenges to conventional therapies due to drug resistance, systemic toxicity, and poor drug targeting efficiency. Biomimetic biomaterials offer unique advantages for precision leukemia therapy, surpassing conventional synthetic materials in biocompatibility, biodegradability, immune evasion, and inherent targeting. These properties enable applications such as hydrogel-encapsulated systems for NK cell culture and platelet-mimetic nanocarriers for targeted drug delivery. This review first elucidates the characteristics of leukemia from microenvironmental, metabolic, and immunoregulatory perspectives. It then summarizes the design principles of biomimetic biomaterials tailored for leukemia therapy. Furthermore, the article details diverse applications of these strategies in anti-leukemic biomaterial platforms. Finally, the challenges and future opportunities for biomimetic anti-leukemia biomaterials are critically discussed across translational, technological, and interdisciplinary dimensions. By providing a comprehensive reference for researchers, this review aims to inspire innovative strategies and accelerate the development of next-generation biomaterials for leukemia treatment.

## 1 Introduction

Leukemia is a common malignant tumour of the blood, accounting for 2.5% of new cancer cases and 3.3% of cancer-related deaths worldwide (Sung et al., 2021). As a non-solid tumour, its metastatic and recurrent nature necessitates therapeutic approaches capable of effectively targeting leukaemic cells infiltrating multiple sites, including the bloodstream, bone marrow, and secondary lymphoid organs. Standard clinical treatments such as chemotherapy, radiotherapy and immunotherapy are frequently constrained by drug resistance, systemic side effects, non-specific drug distribution and high recurrence rates (Devine and Larson, 1994). By contrast, biomimetic materials hold the potential to enhance therapeutic precision while reducing off-target effects. Their design is fundamentally based on utilising natural cells as both inspiration and components. Specifically, cell-based biomimetic materials are engineered systems that achieve targeted therapy by utilising or mimicking the structure, function, and biological behaviour of natural cells. These systems exploit inherent biological advantages such as low immunogenicity, the ability to traverse biological barriers, and specific homing capabilities—qualities often lacking in conventional synthetic drug delivery systems. Compared to non-biological materials facing challenges in clinical translation, biomimetic biomaterials are abundant in source and possess significant potential for large-scale preparation and application. These biomaterials can serve not only as standalone anticancer agents but also as sophisticated carriers for delivering therapeutic drugs. Furthermore, they can be synergistically integrated with standard treatments such as radiotherapy and immunotherapy to enhance overall antitumour efficacy (Yu et al., 2023; Tian et al., 2025). This review first outlines the fundamental characteristics of leukemia, subsequently elaborating on the design principles and specific applications of biomimetic materials for cellular bases used in leukemia treatment. Finally, we summarised and discussed the current challenges and future prospects facing this evolving field.

## 2 Characteristics of leukemia

Leukemia is a hematologic malignancy caused by genetic mutations that drive abnormal progenitor cell proliferation. It is marked by elevated leukocyte levels in the bone marrow, blood, and spleen, disrupting normal hematopoiesis and resulting in anemia, susceptibility to infections, and coagulation disorders (Inaba et al., 2013; Döhner et al., 2015; Speck and Gilliland, 2002). The homeostasis of peripheral circulation relies on the differentiation of bone marrow stem cells into myeloid and lymphoid lineages, ultimately producing functional end-stage blood cells. Acquired genetic and genomic abnormalities drive the malignant transformation of these precursor cells, leading to various leukemia subtypes. The type of leukemia depends on the rate of progression (acute and chronic) versus the type of cells affected (myeloid and lymphoid) and is usually classified as acute myeloid leukemia (AML), chronic myeloid leukemia (CML), acute lymphoblastic leukemia (ALL), and chronic lymphocytic leukemia (CLL). The heterogeneity of all leukemia subtypes arises from two distinct sources. One is the accumulation of leukemic blasts. These rapidly dividing, short-lived immature cells dominate the peripheral blood. The other source is a rare population of leukemia stem cells (LSCs). This quiescent subpopulation possesses self-renewal capacity. LSCs drive disease initiation and promote relapse through their intrinsic resistance to conventional therapies.

### 2.1 Microenvironment of leukemia

**FIGURE 1:**
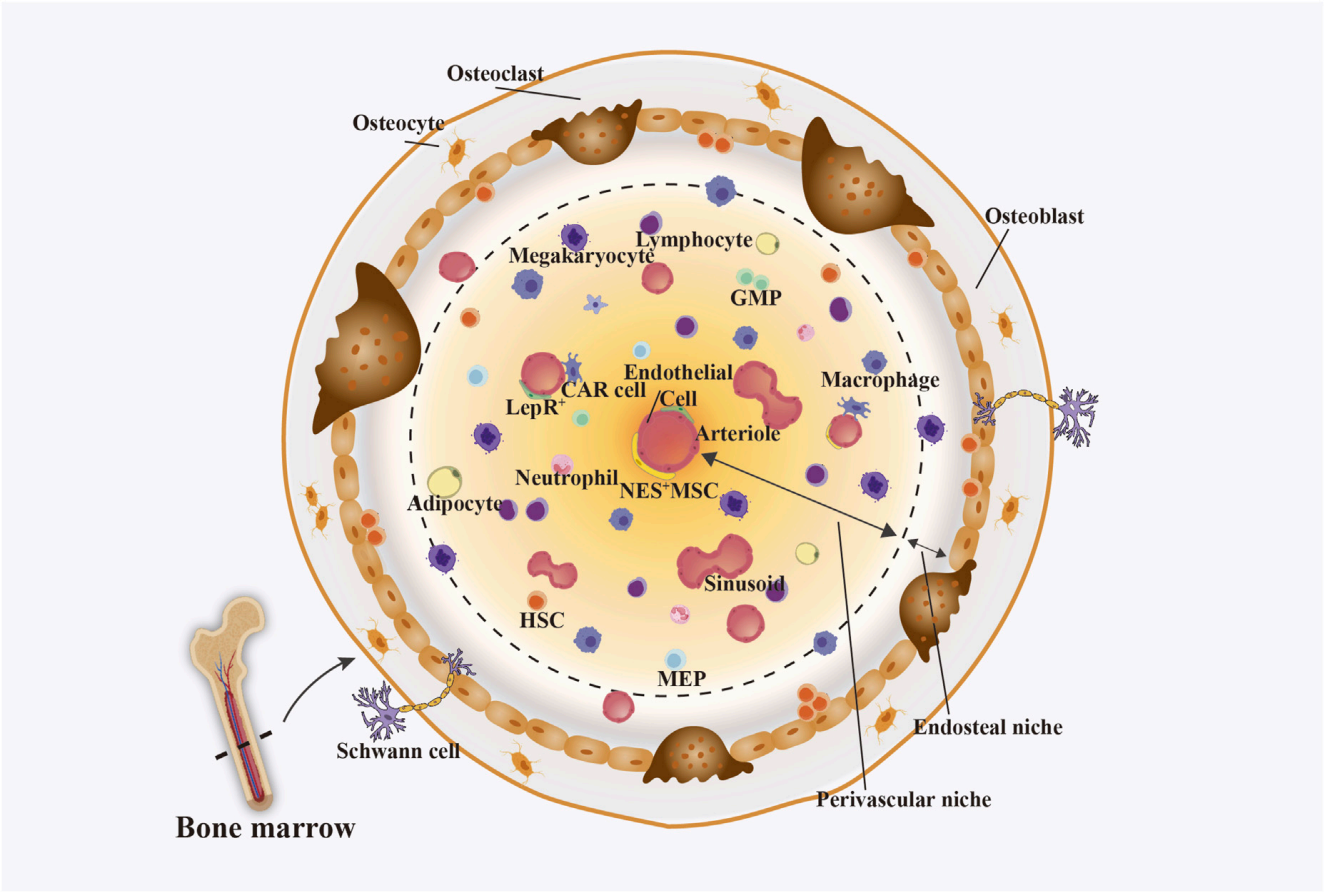
BMM, which consists mainly of endosteal niches and perivascular niches. The endosteal niche contains mainly osteocytes, osteoblasts, osteoclasts, and Schwann cells. The perivascular niche consists mainly of haemocytes, immune cells, endothelial cells, and nestin-expressing mesenchymal stem cells (Nes^+^MSCs). Among them, CXC-chemokine ligand 12 (CXCL12) secreted by endothelial cells, leptinreceptor-expressing perivascular cells (LepR^+^) cells, Nes^+^MSCs, and reticulocytes (CAR) cells in osteoblasts and perivascular niches can control the homing, retention, and repopulation of HSC. Figure created with Adobe Illustrator and biogdp.com.

The bone marrow microenvironment (BMM), as a highly collaborative ecosystem (Figure 1), consists of cellular compartments containing myeloid cells, non-myeloid cells, and non-cellular compartments containing extracellular matrix (ECM), oxygen, and a fluid environment (cytokines, growth factors, chemokines) (Galán-Díez et al., 2018; Shen and Nilsson, 2012). Differentiation, migration, and drug resistance of malignant cells, as well as the development and progression of leukemia, both of which are associated with an abnormal mesenchymal cell composition in the BMM (Kawano et al., 2015). Excessive collagen secretion by stromal cells (e.g., COL1A1) forms a physical barrier preventing the penetration of chemotherapeutic agents and activates the integrin/FAK pathway to promote cancer cell survival and migration (Valkenburg et al., 2018). Lower oxygen concentrations in the BMM were found to be effective in activating the HIF-1α pathway, promoting leukemia cell survival resistance (Wang et al., 2016) and rendering oxygen-dependent chemotherapeutic drugs ineffective while promoting LSCs to enter a dormant state, thereby evading killing by chemotherapeutic drugs (Li Y. et al., 2021). Tumor cells preferentially metabolize glucose via glycolysis even under oxygen-sufficient conditions, a phenomenon termed the “Warburg effect.” This leads to lactate accumulation and acidification of the tumor microenvironment. The resulting acidic extracellular pH alters the ionization of chemotherapeutic drugs, reducing their cellular uptake, while concurrently activating lysosomal enzymes that promote drug damage repair (Wojtkowiak et al., 2011; Barar and Omidi, 2025). Bone marrow stromal cells (BMSC), a core component of BMM, directly or indirectly influence leukemia proliferation, survival and drug resistance by regulating multiple signalling pathways and secreting cytokines, as well as by their own immunomodulatory and anti-apoptotic effects (Świerczek-Lasek et al., 2022; Konopleva and Jordan, 2011). Therefore, the design of specific drug release systems targeting the physicochemical properties of the microenvironment based on the characteristics of BMM, stromal cell dynamic interactions, etc., is an important part of leukemia therapy. Researchers are already developing relevant biomaterials for responsive delivery in such environments (Zahariev et al., 2023; Li et al., 2025). These pH-responsive biomaterials, developed on the basis of the acidic environment prevalent in tumour tissues, achieve multiple modulations of the Warburg effect-driven tumour immunosuppressive microenvironment (TME), enabling more targeted release of therapeutic agents, thus leading to an increase in the efficiency of cancer therapy (Ding et al., 2022). There have also been nanomedicines created through BMSC-derived products guided into the bone marrow, ultimately targeting leukemia cells (Niu et al., 2021).

### 2.2 Metabolic reprogramming and oxidative stress in leukemia

**FIGURE 2:**
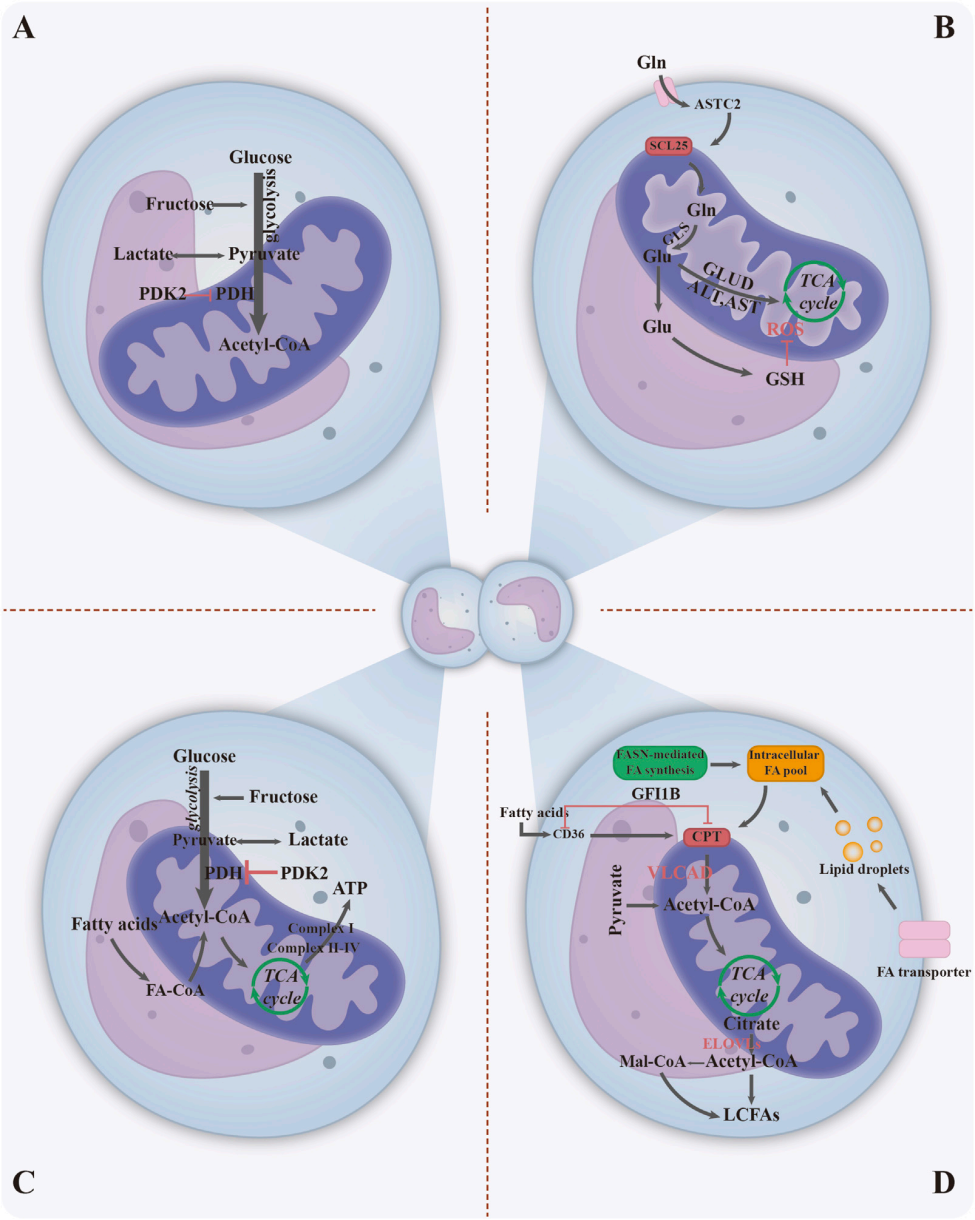
Metabolic reprogramming in leukemia. **(A)** Glycolysis in leukemia cells manifests as elevated carbohydrate metabolism, utilised for energy production and other functions. **(B)** Gln serves both as a carbon and nitrogen source for supplementing TCA cycle intermediates and as a key precursor for synthesising the antioxidant glutathione (GSH). **(C)** In leukemia cells, oxidative phosphorylation within mitochondria is enhanced, leading to increased ATP production for cellular proliferation. **(D)** Fatty acid metabolism and lipid synthesis in leukemia cells: Fatty acid synthase expression is upregulated, accelerating lipid synthesis to support cell division while simultaneously enabling lipid storage to counteract nutritional stress. Abbreviations; Ac-CoA, acetyl-CoA; Gln, glutamine GSH, glutathione FA, fatty acid. Figure created with Adobe Illustrator and biogdp.com.

**FIGURE 3:**
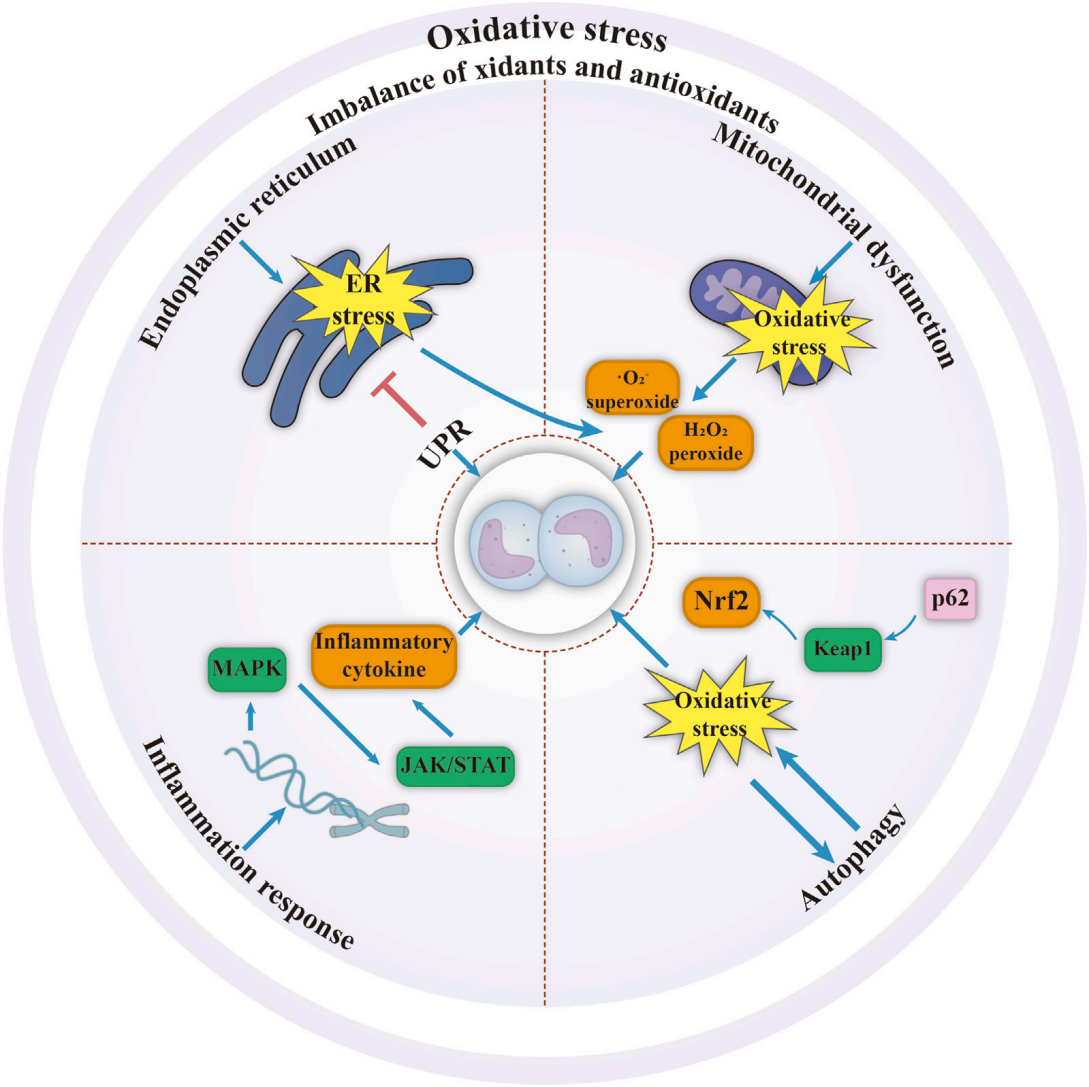
ROS affect leukemia cells by regulating endoplasmic reticulum, mitochondria, inflammatory response, autophagy and other processes (Gurunathan et al., 2020; Wen et al., 2020). Abbreviations; ER: endoplasmic reticulum; JAK: Janus kinase; Keap: Kelch-like ECH-associated protein; MAPK: mitogen-activated protein kinase; Nrf2: nuclear factor, erythrocyte 2 like 2; STAT: signal transducer and activator of transcription; UPR: unfolded protein response. Figure created with Adobe Illustrator.

Metabolic reprogramming in leukemia has the following characteristics (Figure 2): 1) Due to the Warburg effect, leukemia cells rapidly generate ATP via glycolysis even in oxygen-rich conditions, supporting their proliferation and resulting in lactic acid accumulation. Metabolites such as lactate and lipids further act as signaling molecules to promote angiogenesis and immune escape (Figure 2A) (Xu et al., 2024). 2) Glutamine, a carbon and nitrogen source that serves as a complementary tricarboxylic acid cycle (TCA) intermediate and synthesises the antioxidant glutathione, on which AML exhibits a high degree of dependence (Figure 2B) (Dumaswala et al., 2001; Roma et al., 2022). 3) Mitochondrial oxidative phosphorylation is enhanced in AML, promoting ATP production; whereas other types of leukemia may inhibit mitochondrial function, leading to metabolic heterogeneity (Figure 2C) (Zhang et al., 2024). 4) Upregulation of fatty acid synthase expression increases the rate of fatty acid metabolism and accelerates lipid synthesis to meet membrane structural demands, while coping with nutrient stress through lipid storage (Figure 2D) (De Martino et al., 2024). Oxidative stress in leukemia involves a synergistic interaction between excessive intracellular reactive oxygen species (ROS) production, metabolic imbalance due to weakened antioxidant defences and BMM. ROS affect the survival, proliferation and apoptosis of leukemia cells by regulating endoplasmic reticulum stress, mitochondrial dysfunction, inflammatory response, autophagy and other processes (Figure 3) (Gurunathan et al., 2020; Wen et al., 2020). ROS activate multiple signaling pathways and promote cancer progression via transcription factor regulation. ROS accumulation also upregulates PD-L1 expression, suppressing immune cell activity (Zhang et al., 2024). Thus, targeting metabolic pathways to modulate oxidative stress and the microenvironment using biomaterials represents a promising therapeutic strategy.

### 2.3 Leukemia stem cells and treatment resistance

**FIGURE 4:**
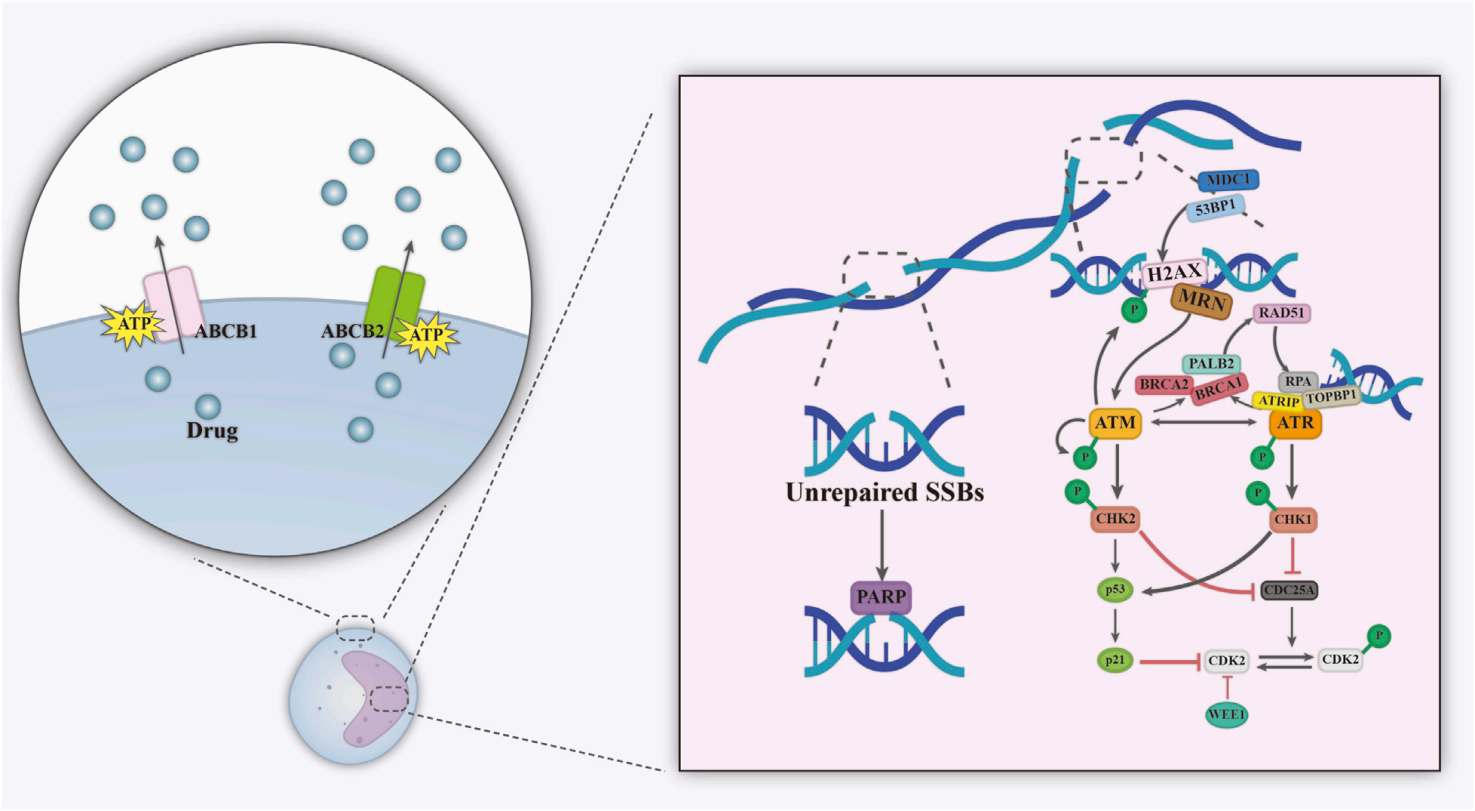
Mechanisms of drug resistance in LSCs, including reverse concentration gradient drug transport and enhanced DNA repair. Abbreviations; ATRIP, ATR-interacting protein; H2AX, histone H2AX; MRN, MRE11, RAD50 and NBS1 complex; RPA, replication protein A; TOPBP1, DNA topoisomerase 2-binding protein. Figure created with Adobe Illustrator.

In contrast to the rapidly dividing and short-lived leukemic blasts, LSCs represent a rare, quiescent subpopulation capable of self-renewal. These cells have unique biological properties that allow them to evade chemotherapy which contributes to relapse and treatment resistance. When unstimulated, LSCs persist in a quiescent G0 phase, suppressing cell cycle progression via p21 and reducing reliance on oxidative phosphorylation (Kim et al., 2025; Iyer et al., 2024). This allows them to evade chemotherapeutic agents (e.g., cytarabine) that depend on cell cycle activity. LSCs have a highly complex resistance mechanism (Figure 4): its high expression of ATP-binding cassette proteins pumps chemotherapeutic drugs against the concentration gradient to the extracellular compartment (Wang et al., 2019). Through mechanisms such as activation of the ataxia telangiectasia mutated/ataxia telangiectasia and Rad3-related (ATM/ATR) pathway, they enhance DNA repair capacity, enabling rapid repair of chemotherapy- or radiotherapy-induced DNA double-strand breaks (Yuan et al., 2014). The high activity of the PARPase enzyme further supports base excision repair (Jaidee et al., 2025), this leads to DNA damage-based therapeutic strategies fail. LSCs can also homing to specific hypoxic regions of the bone marrow via the CXCR4/CXCL12 axis and are tightly connected to stromal cells (Figure 1) (Agarwal et al., 2019). In addition, stromal cells further activated the PI3K/AKT and WNT/β-catenin pathways by secreting stem cell factor (SCF), releasing WNT ligands, and enhancing the stemness and anti-apoptotic capacity of LSCs (Wang YQ. et al., 2024; Xu et al., 2023).

Breakthrough strategies for targeting LSCs focus on dismantling their ecological niche and metabolic barriers. For example, CXCR4-targeted nanocarriers competitively block CXCL12 signalling and drive LSCs into the circulatory system to enhance chemosensitivity (Zeng et al., 2009). Mesenchymal stem cells touch-coated nanofibres (MSCMNF) modified with CXCL12α (CXCR4 receptor) induced LSC migration into the system and promoted apoptosis in LSCs (Ho et al., 2021). Alternatively, biomimetic nanocomposites have been developed to block leukemia homing and eliminate malignant cells from the bone marrow (Kong et al., 2022). Additionally, inhibiting glycolysis key enzymes or combining DNA repair inhibitors synergistically enhances conventional treatment efficacy (Padella et al., 2022; Zhang et al., 2019).

### 2.4 Immune dysregulation and molecular vulnerability in leukemia

**FIGURE 5:**
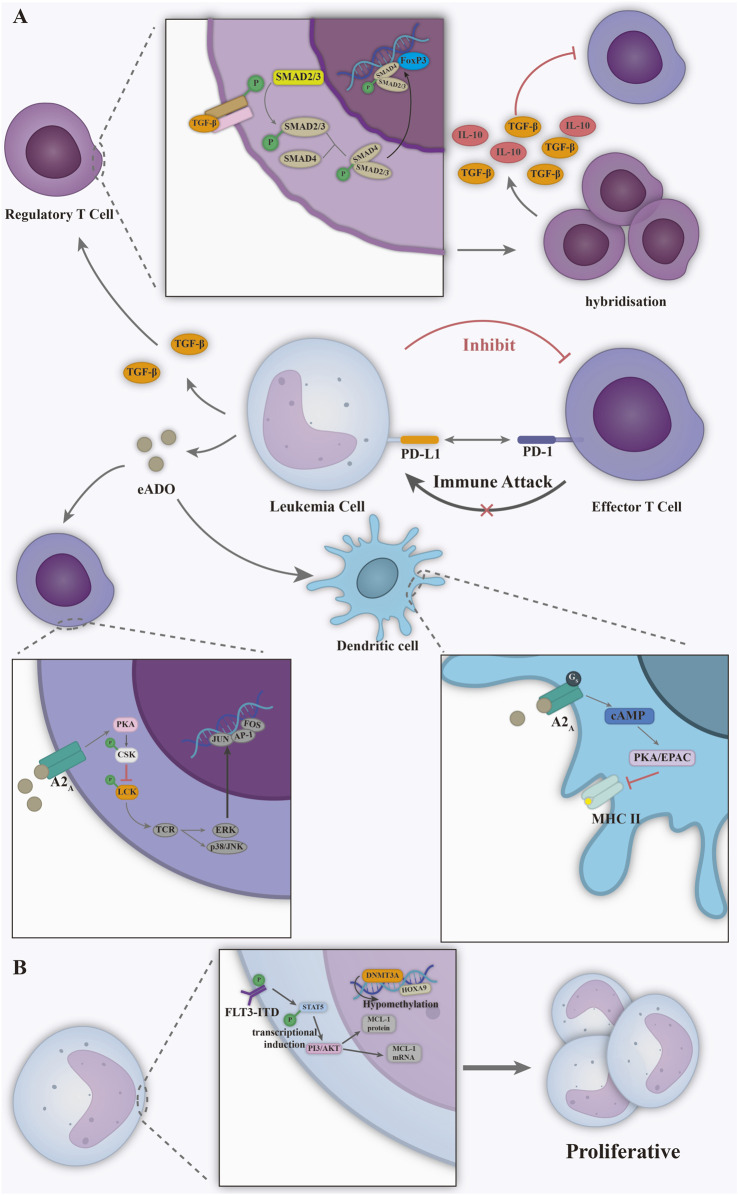
**(A)** Immune dysfunction in leukemia. Leukemia cells bind to the PD-1 receptor on the surface of T cells, while releasing immune factors such as TGF-β and adenosine to achieve immunosuppression of T and DC cells. **(B)** Uncontrolled proliferation of leukemia cells driven by FLT3-ITD mutation, DNMT3A mutation. Figure created with Adobe Illustrator.

The progression of leukemia is also closely linked to immune dysfunction (Figure 5A). Leukemia evades immune surveillance through multiple mechanisms. A key strategy involves PD-L1 overexpression, which binds to PD-1 on T cells, suppressing their proliferation and function, and resulting in immune response paralysis (Balan et al., 2015). Second, leukemia cells remodel the microenvironment by releasing immunosuppressive factors (e.g., TGF-β, adenosine): the TGF-β induces the release of interleukin-10 (IL-10) after the expansion of regulatory T cells (Treg), and the TGF-β factor inhibits proliferation of effector T cells (Teff) (Nishikawa and Koyama, 2021). Adenosine triggers immunosuppression by binding to the A2A receptor on T cells and impairing dendritic cell (DC) antigen presentation, enabling synergistic multicellular immune escape (Vijay et al., 2017; A et al., 2017). Together, these drive the “cold tumour” phenotype. The onset and evolution of leukemia is also dependent on specific molecular vulnerabilities (Figure 5B). Driver mutations like FLT3-ITD promote uncontrolled proliferation via STAT5 activation and represent key targets for tyrosine kinase inhibitors and FLT3-specific therapies (Bystrom and Levis, 2023). At the epigenetic level, DNMT3A mutations lead to extensive genome hypomethylation and activation of oncogenes such as HOXA9 (Sun et al., 2018). Lipid nanoparticles and viral vectors can be antibody-functionalized for targeted drug delivery (Bayraktar and Van Roosbroeck, 2018; Li W. et al., 2021), overcoming the myelosuppression and off-target effects of conventional therapies.

## 3 Biomimetic biomaterial design approaches for leukemia therapy

**FIGURE 6:**
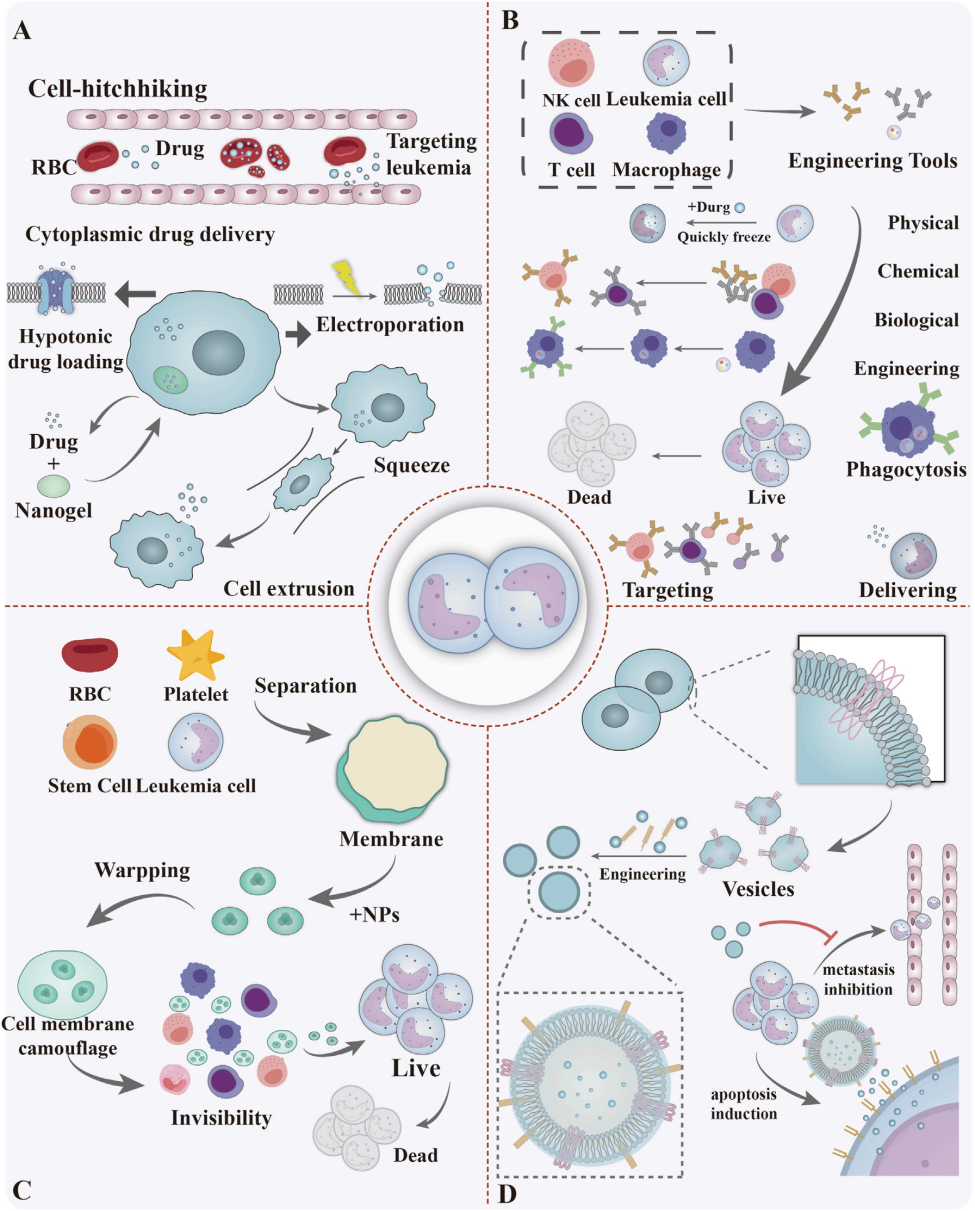
Approaches for the design of biomimetic biomaterial for leukemia therapy, including **(A)** Live cell delivery system: Utilising living cells as carriers for drug delivery. **(B)** Engineered cells, modified via physical, chemical, and biotechnological means, can eliminate leukemia cells by means of specific targeting, direct phagocytosis, and drug delivery. **(C)** Cell-mimetic biomaterials: Enhancing targeted therapeutic efficacy by mimicking natural cellular structures. **(D)** Vesicular cargo delivery systems: Achieving targeted drug delivery using natural cellular vesicles as carriers. Figure created with Adobe Illustrator and biogdp.com.

Various design approaches for biomimetic biomaterial for leukemia therapy are detailed in Figure 6, and their advantages, disadvantages and prospects are detailed in Table 1.

**TABLE 1:** Design approaches, principles, advantages, disadvantages, and prospects of biomimetic biomaterials for leukemia therapy.

Design approaches	Principle/Mechanism	Advantages	Disadvantages	Prospects	Refs
Living cell-mediated delivery vectors	Adsorption of therapeutic agents to the surface of circulating cells Physical encapsulation of therapeutic agents into the cytoplasm of living cells	Enhanced targeting; drug protection; reduced immune clearance; reduced immunogenicity/allergic reaction; improved bioavailability; biological barrier penetration	Risk of cell damage; limited drug loading; stability of carriage difficulty in precise control complexity of carriage process high cost of production and storage	Optimisation of drug carrier shape/surface properties for enhanced adsorption; multifunctional delivery platforms; mild drug delivery technologies; biorthogonal; click chemistry delivery strategies	Xu et al. (2017), Hu et al. (2018), Domenech et al. (2011), Agrawal et al. (2013), Kim et al. (2023), Delehedde et al. (2024), Shah et al. (2020)
Engineered cells	A technique for modifying cells through genetic engineering to give them a specific function or to enhance their original function	Potent and specific; significant efficacy against haematological tumours; high safety profile; potential for allogeneic application.	CRS/ICANS toxicity; antigen escape/loss; insufficient *in vivo* persistence; high production costs insufficient capacity to fight solid tumours and the tumour. Microenvironment	Optimise processes to reduce costs Exploration of *in vivo* CAR programming, CAR dynamic regulation technology; Toxicity mitigation strategies Expanding haematological oncology targets	Wang et al. (2025), Hu et al. (2018), Kailashiya et al. (2019), McArdel et al. (2021), Maude et al. (2014), O’Hear et al. (2015), Schneider et al. (2017), Smith et al. (2017), Hamieh et al. (2019), He et al. (2020), Pham-Danis et al. (2025), Gang et al. (2020), Christodoulou et al. (2021), Albinger et al. (2022), Caruso et al. (2022), Huang et al. (2025), Guoyun et al. (2025), Galea-Lauri et al. (2002), Palma et al. (2008), Rosenblatt et al. (2016), Anguille et al. (2017), Van De Loosdrecht et al. (2018), Janssen et al. (2023), Ci et al. (2020), Garaudé et al. (2024)
Cell-mimetic biomaterials	Synthesis of nanoparticles by encapsulation with natural cell membranes	Homologous/specific targeting Low immunogenicity; multifunctional loading; high safety Long circulation	No standardised and regulated extraction and purification processes Inadequate long-term biocompatibility validation Drug loading efficiency/stability optimisation	Standardised membrane preparation processes Membrane functionalization modification strategies Exploration of cell membrane co-mingling techniques Pharmacodynamic and long-term toxicity assessment	Kong et al. (2022), Chen et al. (2022a), Aryal et al. (2013), John et al. (2022), Harris et al. (2022), Wang et al. (2023), Liu et al. (2022b), Li et al. (2024)
Vesicular cargo delivery systems	Use of natural exosomes/particles as drug/nucleic acid carriers	Natural targeting; low immunogenicity; biological barrier penetration Biocompatibility; natural properties of intercellular communication	Low drug loading efficiency; difficult to standardise production/prepare at scale Controllable *in vivo* behaviour, poor stability Difficulty in isolation and purification	Development of efficient drug-carrying technologies Targeted ligand modification to enhance specificity Synthesis, standardisation and normalisation of production	Hu et al. (2018), Kailashiya et al. (2019), Wu et al. (2023), Usman et al. (2018), Chen et al. (2022b), Xiu et al. (2022), Huang et al. (2022)

### 3.1 Living cell-mediated delivery vectors

Cell hitchhiking utilizes nanoparticles adsorbed onto circulating cells as drug carriers, leveraging their migratory ability to penetrate physiological barriers and achieve targeted delivery (Wang Y. et al., 2024). Positively charged nanoparticles adhere to the anionic phospholipid layer of red blood cells via charge complementarity, enabling efficient delivery of chemotherapeutic drugs to the BMM during circulation (Figure 6A). Despite offering advantages such as enhanced targeting, prolonged circulation time, and reduced immunological clearance, the clinical application of this technology remains constrained by issues including the risk of cellular damage, limited drug loading capacity, and insufficient precision in drug release control. These shortcomings are particularly pronounced in leukaemia treatments requiring high therapeutic concentrations.

Cytoplasmic drug delivery employs physicochemical strategies to encapsulate therapeutic agents into living cells like red blood cells, leukocytes, and platelets. This approach utilizes natural cell membrane barriers to shield drugs from degradation and prolong their circulation time. Cytoplasmic drug loading employs methods such as hypotonic loading, which uses osmotic pressure to enhance membrane permeability for drug uptake; electroporation, which applies an electric field to temporarily permeabilize the membrane; cell extrusion, where physical deformation creates transient pores for delivery; and nanogel encapsulation, which relies on endocytosis for cytoplasmic transport (Hamidi et al., 2011; DeLoach et al., 1991; Magnani et al., 1998; He et al., 2014; Tong et al., 2024) (Figure 6A). Like cell hitchhiking, cytoplasmic drug delivery is hindered by cellular damage risks, low loading efficiency, and complex manufacturing. Demanding storage conditions and high costs further restrict its clinical scalability and broad application.

### 3.2 Engineered cells

Engineered cell therapy for leukemia functionally reprograms cells via genetic engineering to improve tumor targeting and eradication (Figure 6B) (Liu and Hu, 2024). Therapeutic mechanisms include: blocking immunosuppressive signals to counteract microenvironment inhibition; engineering target-recognition constructs for specific antigen binding; and enhancing cell proliferation and stability through genetic modification. Through several steps of cell collection, editing using genetic engineering, *in vitro* expansion and activation, verification of safety and efficacy, and monitoring of efficacy after transfusion, engineered cell therapies are able to mitigate the side effects of normal tissues while providing precise targeting and efficient cytotoxicity to tumour cells. While effective for hematologic malignancies, this therapy is limited by toxicity risks such as CRS and ICANS, as well as antigen escape. In solid tumors, it further faces challenges including off-target effects, poor persistence, and TME suppression (Hong et al., 2020). Recent advances, such as T-cell-specific fusion virus-like particles (T-FVLPmCAR) inspired by HIV fusion mechanisms, have improved mRNA delivery and simplified production, yet issues with low yield and long-term safety remain (Wang et al., 2025).

### 3.3 Cell-mimetic biomaterials

Cell-mimetic biomaterials replicate natural cell structures and functions by modifying or camouflaging cell membranes with biocompatible materials, thereby improving targeting and therapeutic efficacy in drug delivery. Core mechanisms and features include: 1) Natural cell membrane encapsulation of nanoparticles enables immune evasion, prolonged circulation, and homologous targeting via surface receptors. 2) Integration of pH, hypoxia, or ROS responsive materials allows environmental stimulus triggered drug release within the microenvironment. 3) Bionic cells can deliver chemotherapeutic drugs, gene editors, immunomodulators, and combine with photothermal agents for synergistic therapy. 4) High biocompatibility and degradability of the engineered particles jointly ensure *in vivo* safety and functionality (Figure 6C). Cell membrane based targeting therapies reduce off-tumor toxicity and show preclinical promise, but face significant clinical barriers. These include complex extraction, unproven long term biocompatibility, poor metabolic efficiency, batch variability, and inadequate membrane protein preservation. Additional challenges involve low drug loading, limited *in vivo* stability, TME-mediated suppression, and manufacturing scalability issues. These limitations are particularly critical in leukemia due to the complex bone marrow microenvironment and tumor heterogeneity requiring robust and reproducible solutions (Xu et al., 2020; Liu J. et al., 2022).

### 3.4 Vesicular cargo delivery systems

Vesicle cargo delivery systems employ natural extracellular vesicles such as exosomes and microvesicles as core carriers to enable targeted drug and gene delivery by mimicking endogenous intercellular communication mechanisms. Exosomes, typically 30–150 nm in diameter and derived from diverse cell sources, offer low immunogenicity, inherent targeting capacity, and the ability to cross biological barriers. This system operates through multiple mechanisms: exosomes can specifically bind to bone marrow stromal cells to target leukemia stem cells (Li et al., 2023); functional remodeling via electroporation or ligand modification enhances vesicle targeting and therapeutic cargo delivery (Tian et al., 2014); and immune cell-derived exosomes deliver antigens or signaling molecules to directly kill tumor cells or stimulate anti-tumor immunity (Dubois et al., 2023) (Figure 6D). As noted in Table 1, EV therapies face limitations including low drug loading efficiency, absence of standardized isolation and production methods, and unpredictable *in vivo* behavior. Their clinical translation is further hindered by a lack of regulatory guidelines for clinical-grade manufacturing and insufficient understanding of human pharmacokinetics.

## 4 Engineering biomimetic biomaterial for enhancing leukemia therapy

### 4.1 Blood cells

#### 4.1.1 PLTs

Platelets are a potential platform for targeted drug delivery due to their natural targeting, high biocompatibility, ability to be activated by external stimuli for controlled drug release, ability to support multiple drug loads, and excellent disease penetration compared to traditional delivery systems (Zhu et al., 2024). Leukemia cells can activate platelets by secreting substances such as ADP and also inhibit platelet aggregation. On the other hand, platelets promote leukemia cell proliferation and inhibit apoptosis through direct adhesion or release of soluble mediators such as PDGF, PF-4 and VEGF (Bruse et al., 2008).

##### 4.1.1.1 Living PLTs-mediated delivery vectors

**FIGURE 7:**
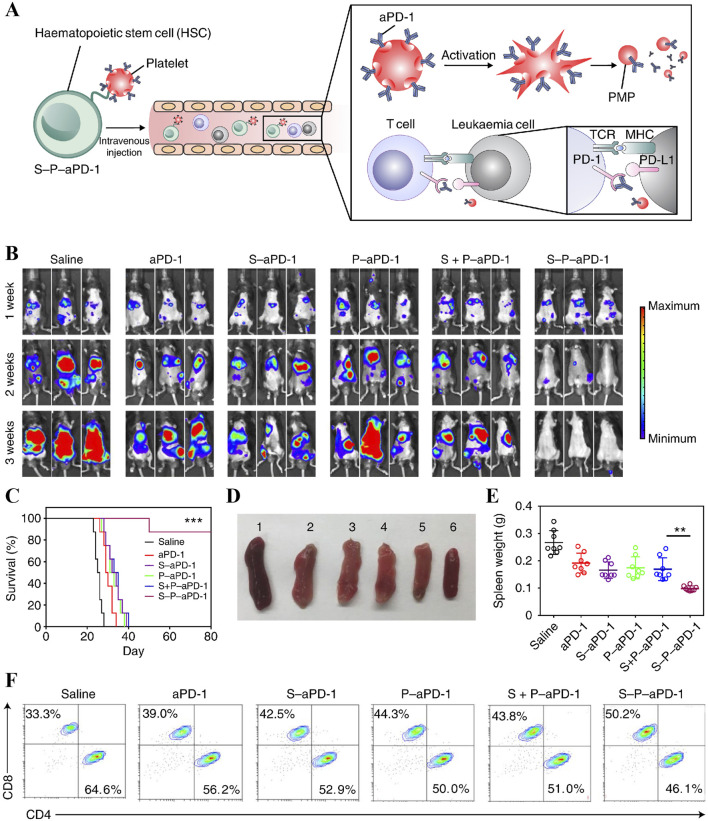
**(A)** Schematic depicting HSC-PLT assembly complex S-P-aPD-1-assisted aPD-1 delivery. **(B)** *In vivo* bioluminescence images of mice after receiving different treatments. **(C)** Survival curves of treated and control mice. **(D)** Spleen morphology of mice after receiving different treatments. **(E)** Mean spleen weights of mice after receiving different treatments. **(F)** Intergroup comparison of CD8^+^ T cells in peripheral blood of mice (Hu et al., 2018).

Natural platelets have been used to load DOX as a potential drug carrier in lymphoma therapy due to their biocompatibility and specific targeting of vascular disease (Xu et al., 2017). However, most of the current data on platelets as a targeted delivery vehicle are derived from single-focal tumour models, quantitative analyses of micrometastases or circulating tumour cell models. Due to the presence of circulating escape system losses and given the dependence of the delivery effect on physical contact, a therapeutic concentration of at least two drug-carrying cells per tumour cell is required for attachment. Therefore, in tumours of the haematopoietic system with a high therapeutic load, such as leukemia and lymphoma, there are practical barriers to the use of platelets as a drug delivery system at the pharmacokinetic level (Cacic et al., 2023). The efficiency of their targeted delivery is limited. Based on this, Hu et al immobilised anti-PD-1 antibody (aPD-1) on the platelet surface by covalent coupling, and then modified platelets (P-aPD-1) were formed by click chemistry with HSCs efficiently coupled to obtain a stable complex S-P-aPD-1 (Figure 7A) (Hu et al., 2018). This system exploits the homing ability of HSCs with platelet piggybacking and drug release function to achieve efficient drug delivery. It inhibited leukemia progression while resisting leukemia cell reattack and achieved good efficacy in animal models (Figures 7B–E), while exploring the mechanism behind it may be the enhancement of T cell immune response (Figure 7F). This novel cell conjugate not only solves the problem of poor bone marrow targeting of anti-PD-1 antibodies in AML therapy, but also mitigates the occurrence of toxic side effects.

##### 4.1.1.2 Engineered PLTs

Platelets in the circulatory system can naturally carry genetic material such as mRNA and function in transcellular communication by releasing platelet microparticles (PMPs). Kailashiya et al developed a “top-down” drug delivery system based on this (PMPDOX) (Kailashiya et al., 2019). The experiments engineered the modification of platelets to efficiently encapsulate adriamycin (DOX) within PMPs, and the use of calcium ion carriers to stimulate the release of PMPDOX from platelets preloaded with the drug, achieving efficient delivery of the target molecule without artificial modification in samples from leukemia patients through a PSGL1 receptor-mediated physiological recognition mechanism. While achieving highly specific killing of leukemia cells, it selectively reduces the extravascular leakage of the drug through the endothelial barrier, effectively solving the problem of off-target toxicity of the drug. The carrier can carry multiple drugs at the same time, has high preparation efficiency and a wide range of material sources, and has a promising future in clinical applications. Modification of platelets by cell engineering techniques to generate PMPs upon activation as aPD-1 delivery vehicles, which in turn act as hubs for immune regulation, mitigating the risk of cytokine surges by local slow release of aPD-1 and targeting specific pathways PD-1/PD-L1 avoiding interference with B-cell related developmental signalling (Hu et al., 2018).

##### 4.1.1.3 PLTs-mimetic biomaterial

**FIGURE 8:**
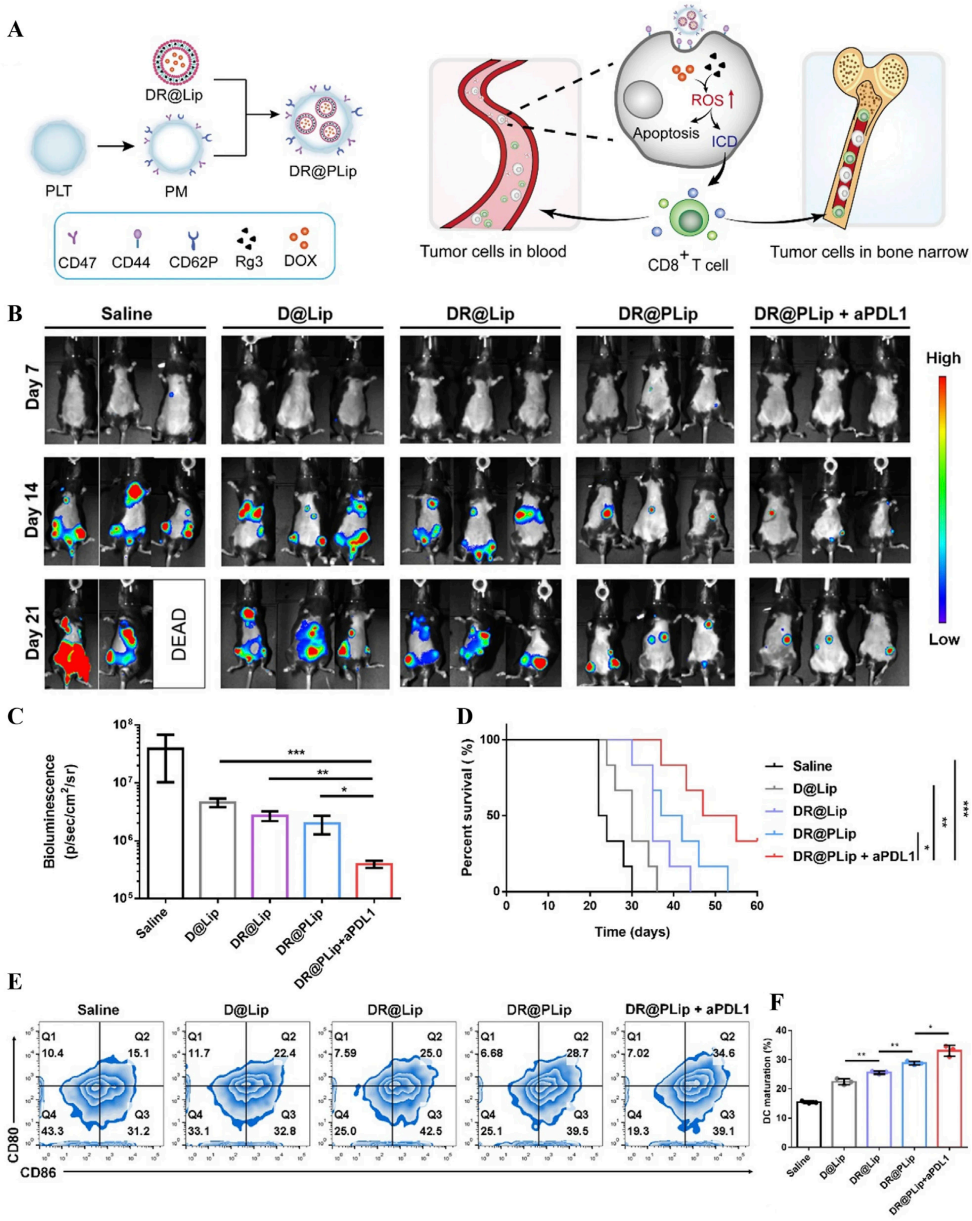
**(A)** Schematic diagram of DR@PLip preparation process and anti-tumour mechanism. **(B)** Biofluorescence images on days 7, 14, and 21 after treatment. **(C)** Biofluorescence intensity on day 22 after treatment. **(D)** Mouse survival curves. **(E)** Mature DC cells in lymph nodes with **(F)** Statistical analysis (Chen M. et al., 2022).

Chemotherapy is the most conventional treatment for acute leukemia in clinical practice, and the “3 + 7” regimen (3 days of adriamycin + 7 days of cytarabine) developed for acute leukemia is widely accepted as the standard of care for achieving a complete cure in the majority of children with a good prognostic risk and those with a moderate to poor prognostic risk (Pelcovits and Niroula, 2020). However, high risk of infection, non-specific distribution of the drug, systemic toxicity and high relapse rate due to drug resistance are its current drawbacks. Platelet membranes express the immunomodulatory protein CD47 on their surface to prevent uptake and also enhance targeting of leukemia cells through P-selectin/CD44 (P-selectin (CD62p)/CD44 interaction) (Hanke et al., 2014; Hu et al., 2015). Based on this, Chen et al developed platelet membrane-camouflaged adriamycin/ginsenoside (DOX/Rg3) co-loaded nanosystems (DR@PLip), which solved the problem of relapse due to poor drug penetration and immunosuppressive microenvironment in AML myeloid lesions through synergistic chemo-immunotherapeutic mechanism (Figure 8A) (Chen M. et al., 2022). PLT membrane (PM)-coated DOX/Rg3 *in vivo* circulation time was increased, multidrug piggybacking enhanced the immune system while targeting and killing leukemia cells, and the direct adhesion properties of PM facilitated leukemia cell capture. Mice treated with DR@PLip + aPDL1 had the weakest biofluorescence intensity in the experiment, suggesting the best treatment efficacy (Figures 8B,C), demonstrating the longest survival time (Figure 8D). Experiments confirmed by *in vivo* immune activation that Rg3 and DOX indirectly promote DC cell maturation and activation of T cells to form an effective immune response (Figures 8E,F).

##### 4.1.1.4 PLTs vesicular cargo delivery systems

PLTs are one of the core components of blood and are heavily used in drug carriers due to their natural tumour targeting properties. PLT extrinsic vesicles (PEVs) are divided into two main types: exosomes (EXOs) and microparticles (MPs). As the most abundant vesicles in blood, PEVs have great potential as biomarkers and drug carriers. Existing drug delivery vehicles such as liposomes and polymers suffer from low bioadaptability, off-target toxicity, and high *in vivo* clearance (De Jong and Borm, 2008). In contrast, PEVs can easily cross the blood-brain barrier and target the TME. Therefore, to address the above issues, the complex S-P-aPD-1 synthesised by the previously mentioned Hu et al contains several design strategies. After engineering PLTs to have the capacity for targeted homing to the bone marrow, leukemia cells in the BMM directly or indirectly activate the PLTs, prompting them to release aPD-1-carrying PMPs and disarming tumour-mediated immunosuppressive effects (Hu et al., 2018). Also applying multiple design strategies were Kailashiya et al who engineered activated PLTs to produce drug-carrying particles, PMPDOX, which utilise PLT membrane proteins to achieve specific targeting with high drug loading, high circulatory stability and better leukocytotoxicity than free drug (Kailashiya et al., 2019). In recent years, Wu et al developed a novel anti-tumour delivery system for PEVs and established a standardised process for the preparation of PEVs from clinical-grade PLTs, which provides a new idea for PLT-based tumour-targeted therapy (Wu et al., 2023). However, PLT production relies on donated blood from healthy donors, making scale-up limited and penetration for solid tumour therapy unproven. The generation of PMPs is dependent on PLT activation, which may lead to the release of pro-inflammatory factors that also require further activation regulation. Balancing the use of PMPs in leukemia therapy with assessing the potential PLT procoagulant and thrombotic risk still requires more research experiments to validate.

#### 4.1.2 RBCs

Compared to other cellular therapies, RBCs have many advantages such as natural biocompatibility, high throughput loading, low immunogenicity, complete biodegradability, long cycle time (120 days), and nucleus-free and non-cancerous. And blood transfusion has a long history of operation experience and safety. RBCs have natural immune escape properties that prevent the downgrading of intracellularly delivered drugs, reducing their immunogenicity, and also use natural markers to attenuate immune responses to their surface-bound reagents.

##### 4.1.2.1 Living RBCs-mediated delivery vectors

L-asparaginase has become a fundamental drug for ALL therapy by precisely targeting metabolic defects in leukemia cells, but suffers from short half-life, poor biocompatibility, allergy and coagulation risks. Domenech et al thus developed erythrocyte-carrying L-asparaginase (GRASPA®) therapy and validated its safety and efficacy in a randomised clinical Phase I/II trial (Domenech et al., 2011). Studies have shown that infusion of GRASPA® significantly reduces the risk of anaphylactic reactions and coagulation disorders and improves the safety of the drug compared to the original formulation. Agrawal et al further suggested that erythrocyte-encapsulated L-asparaginase (RBC-ASNase) therapy prolongs the enzyme half-life via erythrocyte carriers, avoids proteolytic degradation and early hepatic/renal clearance, and is particularly suitable for salvage therapy in patients with aspartate synthetase-deficient AML (Agrawal et al., 2013).

##### 4.1.2.2 Engineered RBCs

Immunotherapies such as embedded antigen receptor T-cell therapy (CAR-T) and immune point inhibitors (CPIs) have an important place in the treatment of leukemia, but cytokine storms with side-effects on immature B-cells increase the need for alternative treatments for them (Jackson et al., 2016; Kingwell, 2017). However, most of the techniques currently used are engineered with erythrocyte vesicles at the core, and the use of whole-cell engineering is not yet mature. McArdel et al developed a genetically engineered erythrocyte (RTX-240) co-expressing 4-1BB ligand and IL-15 fusion protein on its surface, which addresses the problem of insufficient T/NK cell activation and hepatotoxicity in conventional immunotherapy through dual-signalling activation properties (McArdel et al., 2021). In the study, RTX-240 enhanced the cytotoxicity of NK cells against K562-targeted leukemia cells, suggesting the potential application of RTX-240 in activating NK cells to kill leukemia cells. However, development for indications such as AML was ultimately suspended in 2022 due to low overall remission rates and because efficacy did not meet expectations.

##### 4.1.2.3 RBCs-mimetic biomaterial

RBCs are widely used to encapsulate nanomedicines. It is characterised by long *in vivo* circulation time, biodegradability and high biocompatibility (Nguyen et al., 2023). Aryal et al In order to address the problems of short circulation time and macrophage clearance in nanoparticle drug delivery systems, DOX was placed into the core of nanoparticles (NPs) by physical encapsulation and chemical bond coupling, and then encapsulated on the surface with red blood cell membrane (RBCm). The drug has a longer cycling time than conventional polyethylene glycol nanopharmaceuticals (PEG-NPs) and achieves a slow and pH-responsive controlled release of the drug and exhibits higher cytotoxicity than free DOX in AML cells (Aryal et al., 2013).

##### 4.1.2.4 RBCs vesicular cargo delivery systems

**FIGURE 9:**
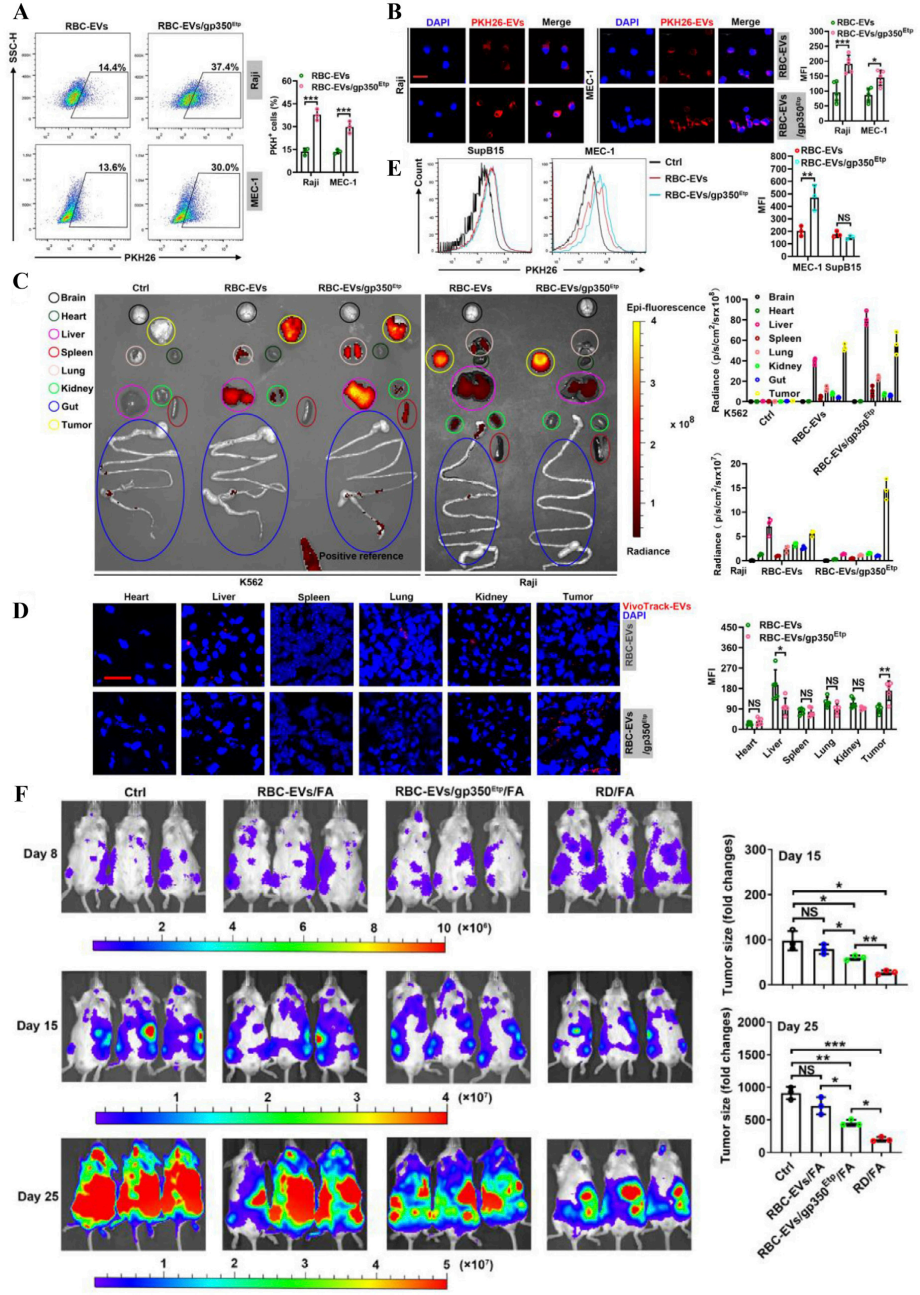
**(A)** Flow cytometry with **(B)** fluorescence microscopy showing strong targeting of RBC-EVs/gp350Etp to CD21^+^ B cells. **(C)** Uptake and **(D)** fluorescence imaging of fluorescently labelled RBC-EV or RBC-EV/gp350Etp in isolated mouse indicated organs with tumours. **(E)** Flow cytometry analysis to detect the mean fluorescence intensity of MEC-1 or SupB15 tumour cells *in vivo* in immunodeficient model NCG mice after injection of fluorescently labelled RBC-EV or RBC-EV/gp350Etp. **(F)** Progression of tumour signal intensity in mice after receiving different treatments (Xiu et al., 2022).

miRNA inhibitors and CRISPR-Cas9 component delivery can specifically target the human genome and are emerging programmable therapies. Viruses and liposomes as existing RNA delivery vehicles suffer from immunogenicity, cytotoxicity and targeting limitations. Waqas et al pioneered a delivery platform based on erythrocyte exosomes (RBC-EVs), confirming the revolutionary potential of RBCEVs as natural RNA delivery vectors for gene therapy (Usman et al., 2018). It ensured efficient co-loading of Cas9 mRNA/gRNA while confirming the stability of *in vitro* delivery, while enabling standardised production of nucleated cell-derived EVs. Chen et al, on the other hand, achieved AML-specific therapy by load-delivering FLT3-ITD and miR-125b via RBC-EVs, forming dual-target synergistic targeting of antisense oligonucleotides (ASOs) (Chen H. et al., 2022). Xiu et al on the other hand, used electroporation to load EBV envelope glycoprotein GP 350-anchored RBC-EVs (RBC-EVs/gp350Etp) with DOX or fludarabine (FA) to selectively induce apoptosis in tumour B cells. Experiments demonstrated strong targeting of gp350Etp to CD21^+^ B cells and less involvement in non-target organs (Figures 9A–D). It was more uptaken by the B-CLL cell line MEC-1 (Figure 9E) and showed more significant tumour suppression in mice (Figure 9F), which improved survival and no systemic toxicity was observed during the period (Xiu et al., 2022). Overall, all these studies demonstrated the potential of EVs in the treatment of haematological malignancies, but further optimisation of their targeting and drug delivery technologies for different targets and delivery strategies is still needed for clinical translation.

### 4.2 Immune cells

#### 4.2.1 T cells

T cells are at the core of both adaptive immunity, triggering direct killing or immunomodulation of leukemia cells through TCR recognition of antigens on the surface of leukemia cells, and the source of malignant transformation in T-cell acute lymphoblastic leukemia (T-ALL). T cells serve the dual role of defender and cause of disease. Its unique antigen-specific recognition ability, long-lasting immune memory function, and target-response integration biological properties have become the cutting-edge focus of cellular biomimetic material design.

##### 4.2.1.1 Engineered T cells

Engineered T-cell technology is active in the treatment of leukemia through genetic modification, modification of cellular physics, chemistry and cell surface engineering. Among them, overdose T-cell therapy has demonstrated strong efficacy in treating haematological tumours by genetically modifying T-cells. The most widely used in leukemia treatment is CAR-T therapy.

**FIGURE 10:**
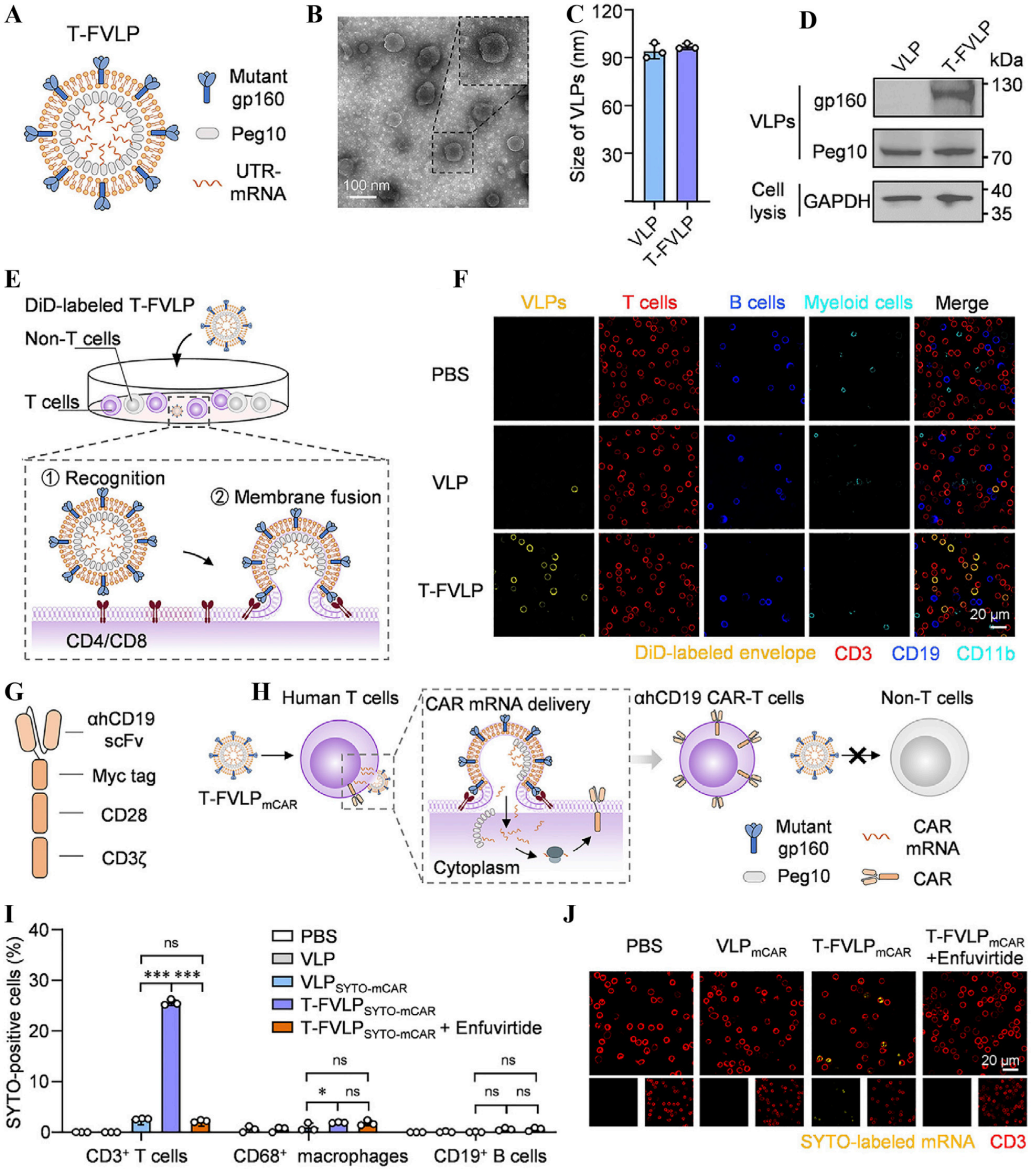
**(A)** Schematic of the T-FVLP structure. **(B)** Transmission electron microscopy image of T-FVLP. **(C)** Size of the T-FVLP. **(D)** T-FVLP proteomic analysis. **(E)** T-FVLP incubated with PBMCs (containing both T cells and non-T cells). **(F)** Confirmation that T-FVLP (yellow, DiD-labelled) specifically fuses with T-cell membranes (red, CD3-PE-labelled) by immunofluorescence imaging. **(G)** Anti-human CD19 (ahCD-19) CAR structural scheme. **(H)** The process of generating ahCD19 CAR-T cells using T-FVLPmCAR. **(I)** Statistics of ahCD19 CAR mRNA delivery to T cells in human PBMCs analysed by flow cytometry. **(J)** Immunofluorescence imaging confirms that T-FVLP (yellow, DiD-labelled) specifically fuses with T-cell membranes (red, CD3-PE-labelled) (Wang et al., 2025).

Engineered T-cell technology has a long history of development. It has shown lasting efficacy in the treatment of leukemia in the last 10 years. The study by Maude et al focused on CD19-targeted CAR-T cells in patients with ALL and demonstrated their remission-inducing ability in refractory disease, providing an important clinical rationale for the subsequent development of CAR-T technology (Maude et al., 2014). O’Hear et al explored the use of CD33-targeted CAR-T cells in AML, reflecting the expansion of CAR-T technology to non-CD19 targets (O’Hear et al., 2015). Schneider’s study, on the other hand, proposes a lentiviral vector design for tandem CD19/CD20 CARs aimed at reducing the risk of leukemia escape mutations through dual antigen targeting (Schneider et al., 2017). The experimental results suggest that tandem CARs may exhibit lower toxicity in high load disease models while efficiently killing tumour cells, but the phenomenon of co-downregulation of target antigens with a new possible escape mechanism was also observed. Smith et al developed an *in situ* T-cell programming technology based on synthetic DNA nanocarriers (Smith et al., 2017) to achieve *in vivo* modification of leukemia-specific T cells via non-viral vectors, reducing the complexity of conventional CAR-T production and enabling optimisation of the delivery technology. Subsequently, Hamieh et al revealed a new mechanism of CAR-T cell “trogocytosis” leading to the escape of tumour antigens (Hamieh et al., 2019). They found that CAR-T cell uptake through membrane proteins reduces the density of antigens in target cells, and put forward the idea that synergistic killing strategies can delay the emergence of drug resistance, which provides a theoretical basis for dual-target CAR design. The Sequentially Tumor-Selected Antibody and Antigen Retrieval (STAR) system developed by He et al innovatively addresses the lack of ideal targets for AML (He et al., 2020). The study screened nanobodies specifically binding to CD13 and TIM3 by STAR technology, and constructed dual-target CAR-T cells with enhanced clearance efficiency for LSCs and reduced toxicity for HSCs in animal models, providing a new option for AML treatment. Pham-Danis et al developed a T-cell junction (LAT)-activated CAR-T (ALA-CART) platform to address the failure of CAR-T cells in low-antigen environments, which significantly enhanced cell persistence and killing of low-antigen tumours by restoring LAT phosphorylation, providing a new approach to overcome the intrinsic defects of CAR-T^92^. Existing CAR-T cell generation techniques are limited by the endocytosis efficiency and endosomal escape ability of nanoparticles. Therefore Wang et al designed a T-cell specific fusion virus-like particle (T-FVLPmCAR) (Wang et al., 2025). This vesicle-sized virus-like particle, composed of mutants gp160 and Peg10 (Figures 10A–D), specifically fused to T-cell membranes when co-incubated with human peripheral blood mononuclear cells (PBMCs) (Figures 10E,F). T-FVLPmCAR bypassed the endocytosis pathway when delivering ahCD19 CAR mRNA (Figure 10G), reprogramming T cells into ahCD19 CAR-T cells in a T cell membrane fusion manner (Figure 10H). The process demonstrated specificity of T-FVLPmCAR delivery (Figure 10I), versus high efficiency of delivery (Figure 10J), but with low yields and insufficient assessment of long-term safety and transfection efficacy.

Cytokine release (CRS) and immune factor-associated neurotoxicity (ICANS) are the biggest challenges facing engineered T-cell technology today, and accordingly, intelligent CAR design with conditional activation or the introduction of suicide switches-iCasp9 suicide genes in combination with a blocking antibody to lower theincidence of severe CRS. In response to drug resistance, multi-target tandeming with epigenetic sensitisers is on the agenda. Engineered T-cells are shifting from “brute force killing” to “precision regulation,” gradually achieving a delicate balance between low toxicity and high efficiency in leukemia treatment.

#### 4.2.2 NK cells

##### 4.2.2.1 Living NK cell-mediated delivery vectors

NK cell-based immunotherapy has been developed for cancer treatment, but traditional intravenous immunotherapy has limitations such as poor tumour targeting and difficulties in *in vivo* expansion (Ahn et al., 2020; Leach et al., 2019; Navin et al., 2020). Therefore, Kim et al proposed a 3D bioprinting-based microporous/macroporous hydrogel encapsulation system for NK cells (Kim et al., 2023). The study used a sodium alginate-gelatin composite hydrogel, in which gelatin dissolves due to heat sensitivity to form micropores that promote NK cell aggregation and enhance their activity and killing ability, while the macroporous structure formed by 3D printing optimises oxygen and nutrient delivery to improve cell survival The system was confirmed to significantly enhance the cytotoxicity of NK92 and epidermal growth factor-specific CAR-NK (zEGFR-CAR-NK) cells against K562 leukemia cells and in an *in vitro* model, providing an appropriate micro-macro-environment for the treatment of NK cells in leukemia and solid tumours, and demonstrating the unlimited possibilities of 3D bioprinting technology in the clinical application of leukemia treatment. Delehedde et al developed imidazolium lipid-based nanoparticles (iCD-Lx/iD-LNP), which encapsulated IL-2 mRNA by microfluidic technology, and successfully enhanced the proliferative capacity and cytotoxicity of NK cells, which was validated in *in vitro* experiments in K562 cells (Delehedde et al., 2024). This study optimised the lipid formulation and mRNA modification strategy, which significantly enhanced the transfection efficiency compared to conventional methods, while reducing the cytotoxicity to the own organism.

##### 4.2.2.2 Engineered NK cells

Compared to the problems faced by CAR-T cells, such as CRS and relapse resistance, CAR-NK cells have received more and more attention from researchers for their advantages of CAR targeting and natural killing, and thus the development of novel targeted immunotherapies. Gang et al developed CD19/33-targeted CAR-ML NK cells by combining memory-like NK cells (ML NK) with CAR engineering technology, which significantly enhanced the killing effect on NK-resistant B-cell lymphomas/leukemias and avoided the toxic side effects common to CAR-T therapy (Gang et al., 2020). Christodoulou et al on the other hand, developed IL-15-secreting CD123 targeting CAR-NK cells and optimised the signalling domains on its surface, which prolonged the *in vivo* survival of CAR-NK cells while enhancing the killing rate of NK cells *in vitro*, but potential toxicity due to systemic IL-15 secretion still needs to be resolved by the development of controlled expression systems (Christodoulou et al., 2021). Albinger’s team developed CAR-NK cells targeting CD33, which were efficiently transduced into peripheral blood NK cells via baboon envelope pseudotyped lentiviral vectors, increasing the expression rate and stability of the CAR, which was demonstrated to significantly reduce leukemia load in an animal model but has not yet been validated for clinical translation (Albinger et al., 2022). Caruso et al on the other hand, focused on the CD123 target and developed a general-purpose CAR-NK therapy based on healthy donor peripheral blood NK cells, which demonstrated 92% clearance of primary AML cells in *in vitro* experiments and proved to have a better safety profile compared to CAR-T cells in a mouse model, although preclinical models suffer from a lack of durability limitation (Caruso et al., 2022). Minimal residual disease (MRD) refers to AML patients with a high relapse rate after initial induction chemotherapy due to the presence of drug-resistant leukemia cells (Schuurhuis et al., 2018). Huang Developed Cord Blood-Derived CAR-NK Cell Therapy Targeting CD33 and Evaluated the Efficacy of Cord Blood-Derived CD33-CAR-NK in a Phase I Clinical Trial (NCT05008575). Six of ten patients with relapsed refractory AML (R/R AML) who met the criteria for efficacy assessment achieved MRD-negative complete remission without severe CRS or graft-versus-host disease (GVHD). It addresses the problems of severe myelosuppression and limited allogeneic application of conventional CAR-T therapy, but long-term efficacy still needs to be validated in larger samples (Huang et al., 2025).

#### 4.2.3 Macrophages

##### 4.2.3.1 Engineered macrophages

**FIGURE 11:**
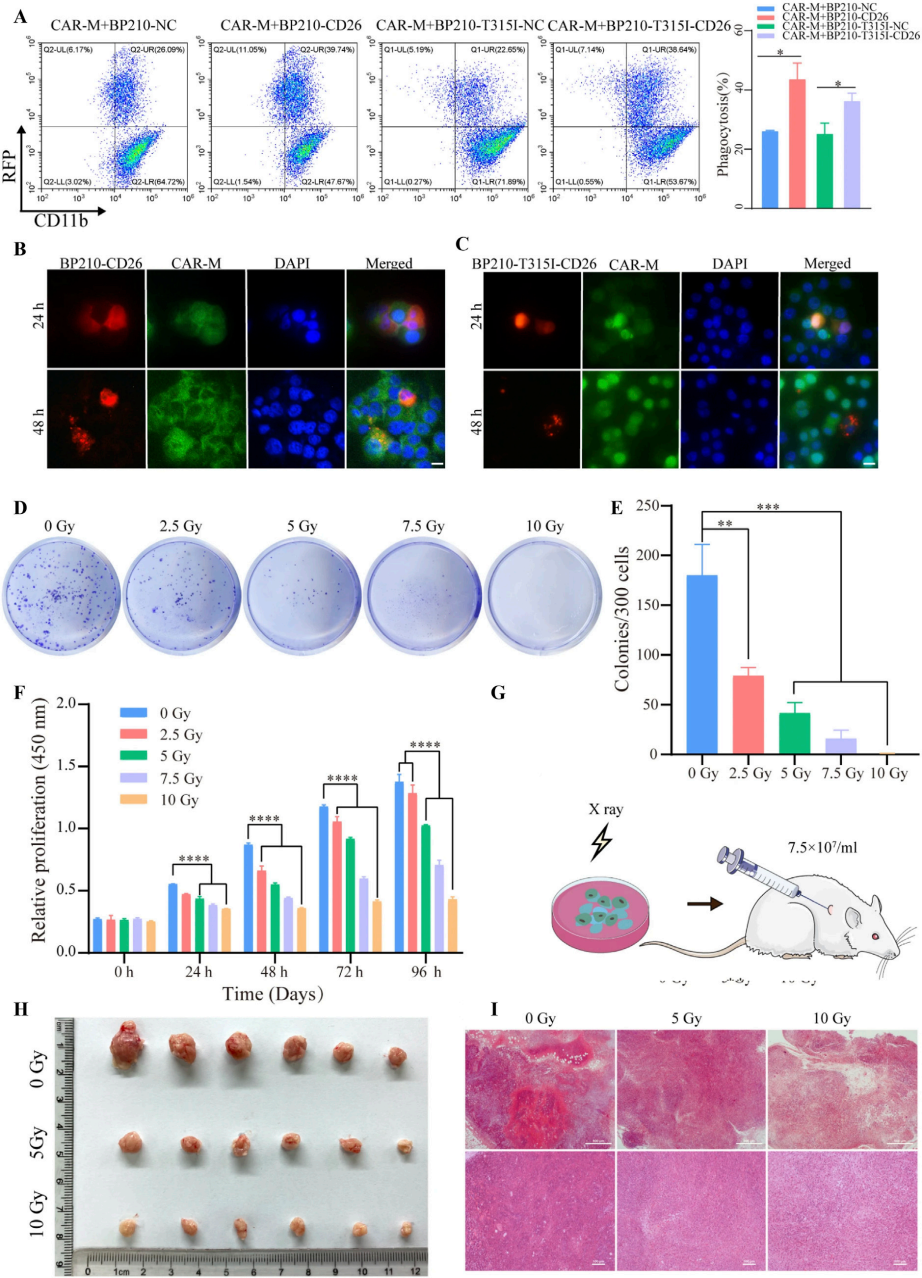
**(A)** Flow cytometry detection of CAR-M phagocytosis of target cells. **(B,C)** Detection of phagocytosis of CD26-positive CML cells (red fluorescence) by CAR-M (green fluorescence). **(D,E)** Proliferative capacity assay of CAR-M after different doses of X-ray irradiation. **(F)** CCK-8 analyses of CAR-M cell proliferation capacity after irradiation. **(G)** *In vivo* safety assessment of CAR-M. **(H)** Tumour images of each group 21 days after CAR-M injection. **(I)** Detection of CAR-M infiltration in tumour tissue by HE staining (Guoyun et al., 2025).

The main challenge in the treatment of CML is currently the resistance of CML-LSCs to tyrosine kinase inhibitors (TKIs), leading to treatment failure and relapse. To address this challenge, Guoyun et al proposed a novel therapy: chimeric antigen receptor macrophage (CAR-M) technology targeting CD26 (Guoyun et al., 2025), aiming to improve therapeutic efficacy by specifically removing CD26-positive CML-LSCs. This study used mouse-derived macrophages to construct a CD26 CAR-M and overexpressed CD26 in the CML cell lines BP210 and BP210-T315I. The study verified the targeted phagocytosis of CAR-M on CD26-positive cells by flow cytometry (Figure 11A) and confocal microscopy (Figures 11B,C). Subsequently, X-rays were applied to eliminate the tumourigenicity of CAR-M (Figures 11D,E), and the safety of CAR-M was verified by CCK-8, clone formation experiments and animal experiments (Figures 11F,G). The results showed that CAR-M exhibited efficient phagocytosis of CD26-positive CML cells (Figures 11H,I), significantly prolonged the survival of CML model mice, and reduced leukemia cell infiltration in the liver, spleen and bone marrow. The study also showed that CAR-M not only has phagocytic ability, but also acts as an antigen-presenting cell to activate anti-tumour immune responses, further enhancing its therapeutic potential. The limitation of this study is that it has only been validated in mouse models and has not yet been translated clinically. Furthermore, although CD26 is highly expressed in CML-LSCs, its distribution in other tissues may lead to potential off-target effects. This study provides new theoretical support for the use of CAR-M in the treatment of haematological malignancies, but further preclinical and clinical trials are needed to optimise its safety and efficacy.

#### 4.2.4 DC cells

##### 4.2.4.1 Living DC cell-mediated delivery vectors

Tumour vaccines are a common immunotherapy used therapeutically for cancer treatment, but extant vaccine strategies are limited by the transient nature of the immune response due to insufficient delivery efficiency. Shah et al develop a novel microporous biomaterial vaccine for AML treatment (Shah et al., 2020). The vaccine successfully induced a strong anti-AML immune response in a mouse model of AML by delivering granulocyte-macrophage colony-stimulating factor (GM-CSF), the Toll-like receptor 9 agonist CpG oligodeoxynucleotide, and leukemia antigens (peptide antigens, cellular lysates, or *in vivo* recruited AML cellular antigens). It was shown that the vaccine significantly increased dendritic cell activation and recruited dendritic cells while slowing the release of immunomodulators, and successfully induced a strong anti-AML immune response in an AML mouse model. The vaccine, which in combination with chemotherapy clears established AML based on blocking AML cell colonisation, remains efficacious in the absence of a definitive vaccine antigen.

##### 4.2.4.2 Engineered DC cells

Engineered dendritic cells are mostly used in the development of leukemia vaccines. The study by Galea-Lauri et al represents an early exploration of dendritic cell (DC) vaccines for the treatment of AML (Galea-Lauri et al., 2002). The team successfully induced specific cytotoxic T-lymphocyte (CTL) responses against AML antigens *in vitro* through innovative dendritic cell-leukemia cell hybrids (DC-leukemia cell hybrids), which provides a theoretical basis for subsequent individualised vaccine development. Palma et al explored the potential of DC vaccines in the treatment of CLL (Palma et al., 2008). The study used apoptotic leukemia cells loaded with DCs to avoid single antigen escape for the purpose of enhancing patients’ anti-tumour immune response, while monocytes were enriched by immunomagnetic beads to achieve clinical-grade mass production. Rosenblatt et al on the other hand, developed an innovative autologous AML-DC hybrid vaccine by physically fusing AML cells with their own DC cells for the first time, and used a sequential vaccination strategy to target MRD after chemotherapy remission (Rosenblatt et al., 2016). It has resulted in a 4-year progression-free survival rate of 71% in AML patients in remission after chemotherapy, making progress on the difficult issues of the tendency to relapse with conventional chemotherapy and the limitations of allogeneic transplantation. Anguille et al efficiently introduced WT1 mRNA into autologous DCs by electroporation, demonstrated for the first time the correlation between WT1-specific CD8^+^ T-cell responses and clinical outcomes, and evaluated the efficacy of a DC vaccine as a post-remission therapy for acute myeloid leukemia (AML) (Anguille et al., 2017). The DCP-001 vaccine developed by Van De Loosdrecht et al is derived from an AML cell line that expresses multiple leukemia-associated antigens, and its pioneering cryopreservation technology enables an off-the-shelf vaccine that breaks through the complexity of autologous vaccine preparation (Van De Loosdrecht et al., 2018). Janssen et al evaluated the long-term efficacy of an allogeneic leukemia-derived dendritic cell vaccine, DCP-001, in patients with high-risk myelodysplastic syndromes (MDS) and AML, and for the first time reported historical data on median survival of up to 1,090 days in high-risk elderly patients (Janssen et al., 2023).

### 4.3 Leukemia cells

#### 4.3.1 Engineered leukemia cells

AML cells can home to the BMM through specific molecular mechanisms. Based on such properties, Ci et al proposed an innovative tumour cell drug delivery system and cancer vaccine strategy based on cryo-shock treatment in their study (Ci et al., 2020). In this study, AML cells were rapidly frozen in liquid nitrogen, causing them to lose their ability to proliferate but retaining an intact cytoskeleton and bone marrow homing properties, resulting in the formation of “Liquid Nitrogen-Treated (LNT) Cells” that were loaded with the chemotherapeutic drug DOX, which resulted in a significant increase in the concentration of the drug in the bone marrow. When used in combination with adjuvants, LNT cells also promote CD8^+^ T-cell responses, achieving a dual therapeutic strategy with both targeted drug delivery and tumour vaccine functions. However, quantitative assessment of the effect of the freezing process on the integrity of cell membrane proteins is still required.

#### 4.3.2 Leukemia cell-mimetic biomaterial

**FIGURE 12:**
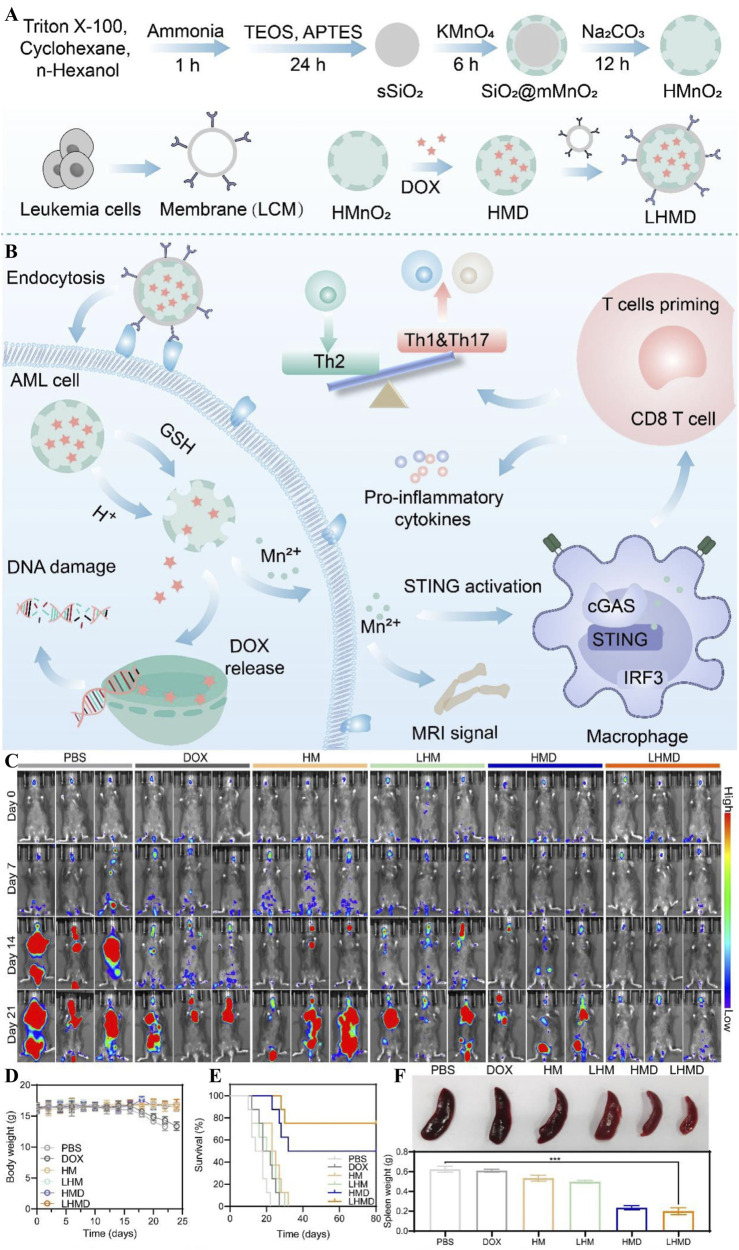
**(A)** Schematic representation of the synthesis process of the nanodrug LHMD. **(B)** Schematic representation of enhanced anti-tumour immunity mediated by chemotherapy and STING pathway in LHMD. **(C)** Bioluminescence images on days 0, 7, 14, and 21 of treatment. **(D)** Body weight changes in AML-loaded mice in different treatment groups. **(E)** Survival curves and **(F)** Spleen pictures and weight curves (Wang et al., 2023).

Cancer vaccines, as a consolidation therapy for AML in MRD, can maintain long-term T-cell immunity by enhancing the delivery of specific antigens by activated antigen-presenting cells (APCs); therefore, the key lies in the formation of specific antigens by APCs and the effective delivery of immunostimulatory adjuvants (Avigan et al., 2018; Fischer et al., 2013). Development of a new technology for AML cell membrane-coated nanoparticle vaccines (AMCNPs) by Zhou et al enables co-delivery of leukemia polyantigens with CpG adjuvants, significantly enhances activation of APCs and T-cell response and establishes a long-lasting immune memory, improving the survival of AML patients (John et al., 2022). For more coherence, Harris et al pioneered the use of the technology of AML cell membrane-encapsulated adriamycin nanoparticles (DOX-MWNPs) applied to targeted chemotherapy (Harris et al., 2022). This technology combines the advantages of pH-responsive release, homologous targeting, and efficient killing to address the non-specific distribution and cardiotoxicity of traditional AML chemotherapeutic agents (He et al., 2019; Zhao and Zhang, 2017) and to provide safer and more targeted therapy for AML patients. Wang et al developed a MnO_2_ nano-loading system (LHMD) for AML cell membrane camouflage. The nanomedicine was based on this using a hollow MnO_2_ carrier (HM) to encapsulate DOX and camouflage it with AML cell membranes (Figure 12A). Its breakdown to Mn^2+^ driven by BMM glutathione (GSH) and an acidic environment activated the STING pathway, which enhanced anti-tumour immunity (Figure 12B) and significantly inhibited leukemia progression (Figure 12C). It ensured biosafety (Figure 12D) while prolonging the survival time of mice (Figure 12E) and alleviating splenomegaly (Figure 8F), achieving synergistic chemo-immune therapy (Wang et al., 2023).

#### 4.3.3 Leukemia cells vesicular cargo delivery systems

EVs deliver loaded drugs to target cells during fusion with the target cell membrane or phagocytic internalisation (Ratajczak and Ratajczak, 2020). A variety of drugs such as DOX, paclitaxel and imatinib can be efficiently loaded into EVs by freeze-thawing, electroporation, ultrasound, extrusion and incubation, reflecting the potential of exosomal delivery in leukemia therapy (Lamichhane et al., 2016; Saari et al., 2015; Patil et al., 2020; Agarwal et al., 2020; Hekmatirad et al., 2021). EVs can also cross the blood-brain barrier, making them viable as drug carriers for CNS leukemia that is not responsive to systemic chemotherapy. Therefore, Huang et al first downregulated PD-L1 expression in ALL-derived exosomes (LEX) by lentiviral shRNA, breaking the natural immunosuppressive barrier of exosomes (Huang et al., 2022). They found that the modified engineered exosome (LEXPD-L1si) significantly enhanced dendritic cell maturation and T-cell activation, achieving dual-pathway synergy, and demonstrated both preventive-cum-therapeutic effects in a mouse model. This study complements the limitations of existing CAR-T therapies and antibody drugs and provides new ideas for overcoming the natural immunosuppressive properties of exosomes.

### 4.4 Stem cells

#### 4.4.1 Engineered stem cells

As a highly effective treatment for widely metastatic haematological tumours, the standard of care for haematopoietic stem cell transplantation (HSCT) is limited by non-targeted chemotherapy and selective eradication of tumour cells after HSCT treatment (Copelan, 2006). Antigen-specific cell depletion therapies have the ability to specifically eliminate diseased cells, but their therapeutic environment is limited by complex target selection and antigen expression in specific cell populations (June and Sadelain, 2018; Perna et al., 2017). Garaudé et al developed an engineered modification of the CD45 epitope based on base editing technology (ABE8e) (Garaudé et al., 2024). It uses an antibody-coupled drug (ADC, CIM053-SG3376) to the pan-hematopoietic marker CD45 to clear the entire haematopoietic system, while introducing the CD45K352E/G mutation in transplanted HSCs via ABE8e to give them ADC resistance. The study achieved selective clearance of haematological malignancies and preserved healthy haematopoiesis. This study provides a paradigm shift in haematological oncology treatment through the strategy of “whole-system clearance + precise reconstruction,” but its clinical translation requires further optimisation of the editing efficiency and extended model validation.

#### 4.4.2 Stem cell-mimetic biomaterial

**FIGURE 13:**
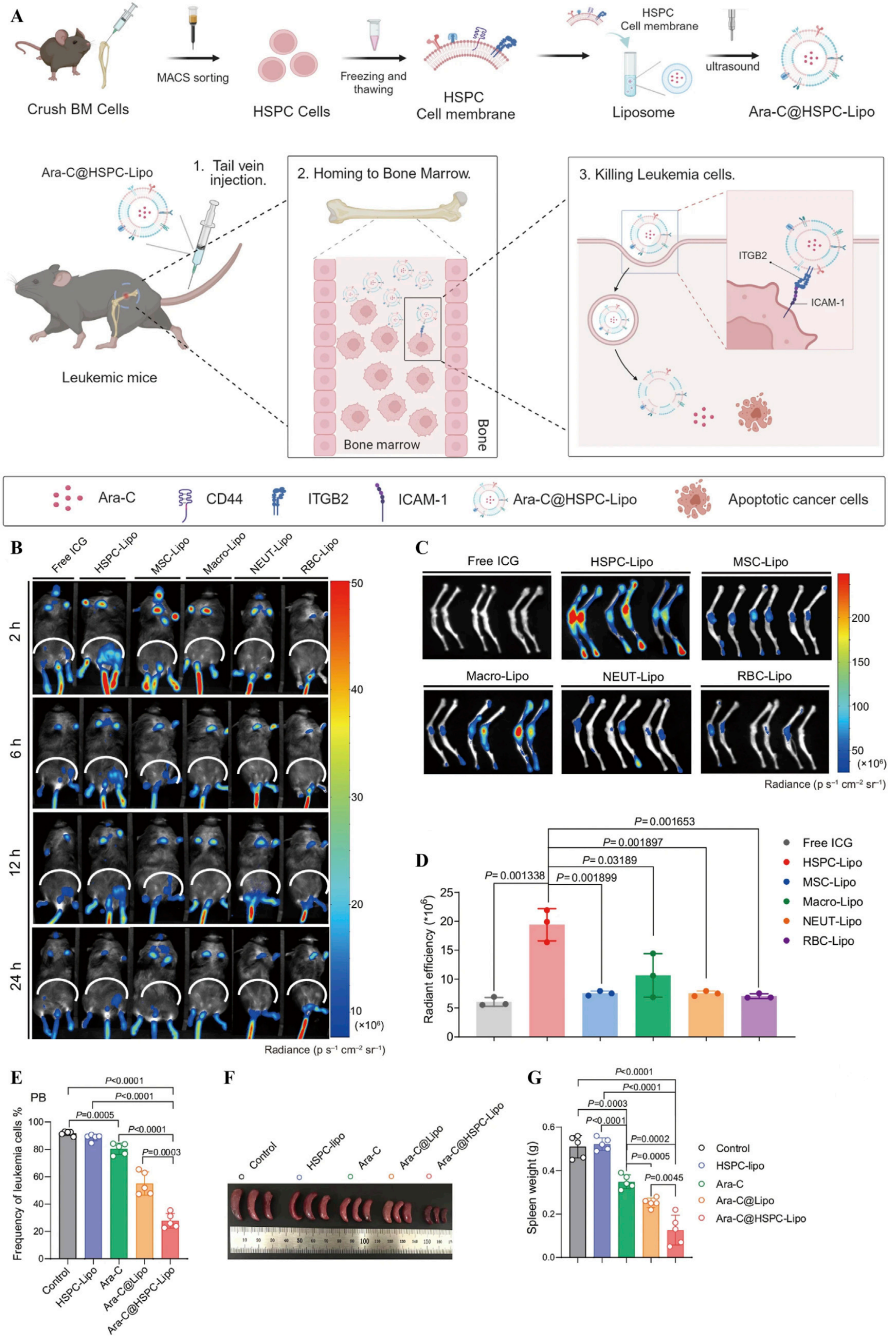
**(A)** Schematic diagram of the process of haematopoietic stem progenitor cell membrane-encapsulated vesicle (HSPC-Lipo) preparation and treatment of leukemia. **(B)** Biofluorescence images at 2, 6, 12, and 24 h after injection. **(C)** Fluorescent images of mouse tibia and femur after 24 h **(D)** Quantitative analysis of different liposomes in mouse tibia and femur. **(E)** Quantitative analysis of peripheral blood leukemia cells. **(F)** Images of spleens from treated mice with **(G)** spleen weights (Li et al., 2024).

The stem cell membrane surface contains a large number of molecular recognition sections that interact with tumour cells and have a natural tumour affinity, making them potentially valuable in leukemia therapy (Gao et al., 2016a; Gao et al., 2016b). Kong et al constructed a biomimetic nanocomposite (PFOB@PLGA@Pt@DOX-CM) formed by bone marrow stromal cell membrane (MS-5) encapsulated with perfluorobromooctane (PFOB), poly(lactic acid)-glycolic acid copolymer (PLGA), platinum nanoparticle enzyme (Pt), and DOX (Kong et al., 2022). The material integrates the triple mechanism of chemotherapeutic drugs, Pt cascade catalysis by platinum nano-enzymes and CXCR4 antagonism to achieve synergistic treatment of AML, while mimicking the homing property to effectively remove residual leukemia cells. In addition to this, it can also block the CXCR4/CXCL12 axis by antagonising CXCR4 and ultimately preventing leukemia cells from homing to the bone marrow/liver and spleen, solving the challenge of organ infiltration blockade in AML treatment. Liu et al proposed a novel macrophage immune reconstitution strategy to treat leukemia by targeting and modulating macrophage phenotype through stem cell mimetic liposomes (sLipo leva) loaded with levamisole (levamisole) (Liu R. et al., 2022). In this study, the integration of mesenchymal stem cells (MSCs) membranes empowered liposomes with the ability to actively target macrophages, while directly inducing macrophage polarisation to M1-type and indirectly activating T cells to synergistically kill leukemia cells, which demonstrated highly effective anti-tumour activity and a good safety profile in a leukemia mouse model. Li et al on the other hand, constructed HSPC-Lipo, a bionic delivery system, by extracting the cell membrane of cord blood haematopoietic stem/progenitor cells (HSPCs) and fusing it with synthetic liposomes (Li et al., 2024). This system fused HSPC membrane CD44 with ITGB2 (Figure 13A) to achieve a dual targeting mechanism of bone marrow-targeted homing (Figures 13B,C) and ITGB2-ICAM-1 with higher targeting efficiency (Figure 13D). It achieves a slow release of cytarabine via a liposomal drug-carrying system, which ultimately reduces the frequency of leukemia cells in the peripheral blood of mice (Figure 13E), reduces spleen weight (Figures 13F,G), and exemplifies leukemia bone marrow-specific drug delivery.

## 5 Conclusions and outlooks

**FIGURE 14:**
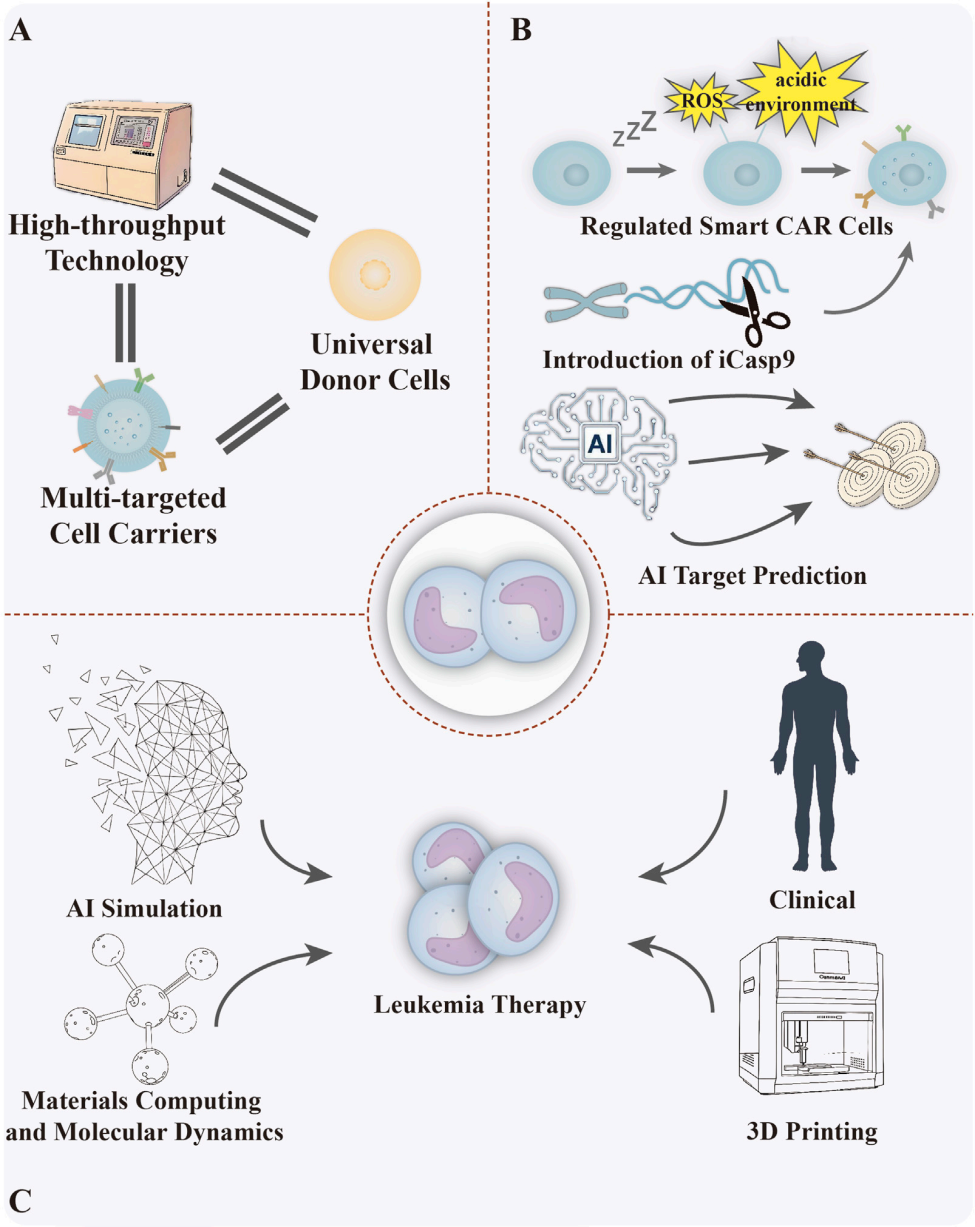
Advancing biomimetic biomaterials for leukemia at three levels. **(A)** Cellular level: high-throughput technology, universal donor cells, multi-targeted cell carriers. **(B)** Therapeutic level: tunable smart CAR cells, introduction of suicide genes, AI target prediction. **(C)** Multi-disciplinary application level: AI dataset construction, molecular material science, clinical medicine, 3D printing technology. Figure created with Adobe Illustrator and biogdp.com.

As an emerging field in leukemia therapy, biomimetic biomaterials encompass a wide range of strategies from live cell-mediated carriers to engineered cell and vesicle systems. Living cell-mediated systems are naturally targeted and biocompatible, but face significant challenges in standardisation, scale-up production and precise control of drug release. In contrast, cytomimetic biomaterials recapitulate important surface functions such as immune escape and homing, yet long-term biocompatibility of membrane separations and consistency between batches remain major hurdles at present. CAR-T and CAR-NK cells, as representatives of engineered cell therapies, have demonstrated unparalleled clinical efficacy in specific haematological malignancies, but are hampered by complex manufacturing processes, high costs and severe immune-related toxicity. Vesicle systems, as natural delivery vehicles with low immunogenicity and the ability to cross biological barriers, are plagued by low drug-loading efficiency, lack of standardised isolation protocols and unpredictable *in vivo* pharmacokinetics. Despite the advantages and disadvantages of each of these strategies, their clinical translation faces many common challenges. In order to systematically advance such materials from experimentation to practice, we further propose translational pathways and solutions at multiple levels (Figure 14).

The stability and safety of cell products are primarily challenged by batch-to-batch variability in biomaterials and the presence of potential foreign substances, which has delayed FDA approval and the translation process from laboratory to clinical application (McKee and Chaudhry, 2017). Secondly, investigating the regulatory mechanisms by which biomaterials influence cellular behaviour during cell production will facilitate the precise selection of biomaterials. Building upon this foundation, further research is required to determine how to select suitable biomaterials that more closely mimic the ecological niche of specific cells. Cell therapy is undergoing continuous advancement, with efforts focused on establishing standardised and regulated production processes, designing carriers with extended circulation, multi-targeted and multi-pathway capabilities, and developing universal donor cells alongside *in vivo* expansion techniques to address quantitative challenges (Figure 14A). Furthermore, ethical concerns associated with cell sources may be addressed through cellular dedifferentiation, transdifferentiation, and the utilisation of umbilical cord blood. The progressive advancement in establishing professional standards and regulations for cell products, conducting safety assessments, and researching regulatory mechanisms is driving the widespread clinical application of biomimetic biomaterials. To address the challenges of tumour heterogeneity and therapeutic resistance, cutting-edge research is driving the evolution of leukemia treatment towards intelligent and multifunctional approaches. For instance, novel diagnostic biomaterials can be engineered using mRNA vaccine technology by designing mRNA sequences for magnetic resonance imaging probes alongside fluorescent protein coding, enabling specific tumour labelling and providing new tools for precision imaging (Younis et al., 2022). Smart CAR technology achieves dynamic regulation of CAR-T activity by incorporating light-controlled/small-molecule switches or components responsive to reactive oxygen species (ROS) and acidic environments. Concurrently, the integration of the iCasp9 suicide gene enhances therapeutic controllability and safety. To further enhance targeting precision, we employ multi-omics analysis and artificial intelligence (AI) to conduct in-depth exploration of the specific molecular characteristics of LSCs, thereby identifying more effective and novel therapeutic targets (Figure 14B). In the field of drug delivery, nanorobots with precision and multifunctionality offer a revolutionary platform for drug synthesis and targeted delivery (Cheng et al., 2024).

More importantly, as experimental datasets continue to expand, researchers face constraints in effectively acquiring, analysing, interpreting and managing them, thereby hindering the rational design and high-throughput development of leukemia treatment materials. This challenge is effectively mitigated through an integrated strategy combining artificial intelligence and big data analytics with diverse material databases, enabling data mining and rapid screening of large-scale datasets. The integration of molecular materials science with 3D printing technology enables cross-disciplinary technological convergence (Figure 14C). Beyond screening novel therapeutic targets and mining experimental data, AI has also demonstrated remarkable capabilities in clinical trial simulation. Eckardt et al. constructed a clinical-genomic multimodal synthetic dataset for AML patients using an AI-generated model (Eckardt et al., 2024). This synthetic data, generated from real-world data, closely mimics patient heterogeneity while maintaining privacy and security. It significantly reduces both economic and time costs while mitigating ethical constraints. However, it remains reliant on simulations and extrapolations of existing data distributions, and its generalisation capabilities are questionable. Concurrently, AI’s explanations for its decision-making processes may be influenced by spurious features unrelated to biological mechanisms. This inherent ambiguity prevents it from directly guiding the design of biomaterials. Moreover, while synthetic data can safeguard privacy, new regulatory standards are required to define its boundaries of validity and reliability within virtual clinical trials. Artificial intelligence and big data analytics offer a powerful new paradigm for research into leukemia biomaterials, though they currently serve only as supplementary tools to traditional biomedical research. By constructing multi-centre, high-quality datasets, fostering the integration of fundamental research with algorithm development, and establishing a dynamic assessment and approval framework for AI technologies, we can transform technological potential into viable clinical solutions.
